# Life in the slow lane; biogeochemistry of biodegraded petroleum containing reservoirs and implications for energy recovery and carbon management

**DOI:** 10.3389/fmicb.2014.00566

**Published:** 2014-11-11

**Authors:** Ian M. Head, Neil D. Gray, Stephen R. Larter

**Affiliations:** ^1^School of Civil Engineering and Geosciences, Newcastle UniversityNewcastle upon Tyne, UK; ^2^Petroleum Reservoir Group, Department of Geoscience, University of CalgaryCalgary, AB, Canada

**Keywords:** biogeochemistry, oil reservoirs, microbial ecology, energy, hydrocarbon biodegradation

## Abstract

Our understanding of the processes underlying the formation of heavy oil has been transformed in the last decade. The process was once thought to be driven by oxygen delivered to deep petroleum reservoirs by meteoric water. This paradigm has been replaced by a view that the process is anaerobic and frequently associated with methanogenic hydrocarbon degradation. The thermal history of a reservoir exerts a fundamental control on the occurrence of biodegraded petroleum, and microbial activity is focused at the base of the oil column in the oil water transition zone, that represents a hotspot in the petroleum reservoir biome. Here we present a synthesis of new and existing microbiological, geochemical, and biogeochemical data that expands our view of the processes that regulate deep life in petroleum reservoir ecosystems and highlights interactions of a range of biotic and abiotic factors that determine whether petroleum is likely to be biodegraded *in situ*, with important consequences for oil exploration and production. Specifically we propose that the salinity of reservoir formation waters exerts a key control on the occurrence of biodegraded heavy oil reservoirs and introduce the concept of palaeopickling. We also evaluate the interaction between temperature and salinity to explain the occurrence of non-degraded oil in reservoirs where the temperature has not reached the 80–90°C required for palaeopasteurization. In addition we evaluate several hypotheses that might explain the occurrence of organisms conventionally considered to be aerobic, in nominally anoxic petroleum reservoir habitats. Finally we discuss the role of microbial processes for energy recovery as we make the transition from fossil fuel reliance, and how these fit within the broader socioeconomic landscape of energy futures.

## An introduction to heavy oil systems

Estimated global bitumen and heavy-oil resources are around 5.6 trillion barrels (bbl), mostly occurring in the western hemisphere. These resources, dominate the world petroleum inventory, and are increasingly being developed as light oil reserves deplete (Hein et al., 2013). This development has driven applied research into more efficient production, and fundamental research into the biogeochemical origin of these enigmatic large-scale oil accumulations. Much of the enabling technical developments have occurred in the largest bitumen and heavy-oil fields of the Canadian oil sands, the Orinoco Heavy Oil belt of Venezuela, the heavy oil on the North Slope of Alaska and the heavy-oil fields of California. Both applied and fundamental studies, are informed by understanding the microbial processes that have acted on, what began as, more conventional oil and gas. In some cases over geological and, in other cases, more recent timescales.

Heavy oil and bitumen have lower hydrogen and higher carbon contents and hence lower energy content than “conventional,” oil and recovery often involves higher energy investment and associated carbon dioxide emissions. Increased oil production from these deposits thus, rightly, raises concerns from society and is now a major political and environmental issue. Perhaps nothing better illustrates the enigmatic and controversial nature of the increasing development of biodegraded oil resources, than the development of Western Canada's oil sands, where output from bitumen reserves, the world's third largest proven crude oil deposit, is expected to climb from around 1.8 million barrels/day in 2012 to 2.3 million barrels/day by 2015, rising to 5.2 million barrels/day by 2030.

### API gravity the standard measure of heavy oil

Heavy oil industry terminology is inconsistent and confusing. Based on the oil density-based industry standard yardstick of oil quality—API gravity, many of the “extra heavy oils” of Venezuela would be considered “oil sands” in Canada or “tar sands” in the United States.

API gravity [in degrees] = [141.5/specific gravity at 60F]−131.5)

Heavy oil is defined as oil with 10–20 degrees API and a viscosity of more than 100 centipoise (cP). Bitumen includes extra heavy oil as well as oil in oil sands, with less than 10 degrees API and viscosity of more than 10,000 cP. The main distinction is that the high viscosity of “bitumen,” prevents it from flowing to a wellbore under *in-situ* reservoir conditions, whereas heavy oils will flow. Heavy oil and bitumen can be regarded as part of a continuum of heavily to severely biodegraded oil (Hein et al., 2013).

### Geological settings and properties of heavy oil and heavy oil reservoirs

Although, heavy oil can be found in all basin styles and sizes, from narrow rift basins to the largest sedimentary basins on Earth, most of the largest heavy oil and oil sands deposits are found in large foreland basins adjacent to orogenic belts, with large source rock kitchens charging large, shallow, cool, reservoirs at the basin flank susceptible to severe *in-situ* biodegradation (Creaney et al., 1994; Adams et al., 2013a). The world's largest oil-sand deposit, located in western Canada, is reservoired in Lower Cretaceous sandstone deposits in a basin adjacent to the Canadian Rocky mountains (a foreland basin) (Head et al., 2003; Adams et al., 2004, 2013a; Larter et al., 2006; Larter and Head, 2014). Petroleum was derived principally from marine shale source rocks with the petroleum migrating eastward several hundred kilometers to accumulate and biodegrade on northeastern margins of the basin. The main phase of accumulation was around 84–55 Ma ago (Adams et al., 2013a; Tozer et al., 2014). The petroleum accumulated in tidal-controlled river and estuarine sediments. Oil was similarly accumulated in foreland basin settings in the Oficina Formation in Venezuela, another major heavy-oil resource.

The primary control on oil composition and viscosity in heavy oil and bitumen containing reservoirs, is in-reservoir, anaerobic biodegradation (Head et al., 2003; Larter et al., 2008). The mechanisms, control, and consequences of this are discussed in detail later in this manuscript. Alberta Lower Cretaceous reservoirs range from 38°API (light oil) in the barely biodegraded oil pools west of the Peace River oil sands, to 6° API (severely biodegraded bitumen) in eastern Athabasca oil sands with even lower values in the most degraded bitumens, found in karsted Grosmont carbonate reservoirs underlying the oil sands. Oil sulfur contents range from 1 to >10 wt%, with the western Peace River oil sands having the highest values. Variability correlates roughly to levels of oil biodegradation which broadly increase from west to east and from south to north (Adams et al., 2006).

The impact of the deep biosphere on petroleum composition is significant since biodegradation affects oil composition which in turn affects fluid flow properties (viscosity), and oil pricing (API gravity), both factors with enormous economic and environmental impact. Oil compositional gradients and resulting vertical and lateral oil viscosity variations (discussed below) are common on both reservoir thickness (tens of meters) and field scales (kilometers) and are a defining characteristic of heavy oilfields. Such gradients in a minority of heavy oilfields can certainly be produced by restricted vertical mixing and by density stratification of an evolving oil charge, as originally suggested by Khavari-Khorasani et al. (1998) and more recently by Stainford (2004). Importantly it is in-reservoir oil biodegradation that substantially produces the systematic compositional gradients seen in heavy oilfields (Larter et al., 2003). Furthermore, continuous vertical compositional gradients in the oil columns document episodic degradation for many millions of years, suggesting that the timescales of oilfield degradation and petroleum charging are similar (Larter et al., 2003). Gradients in chemical composition of oil commonly seen in heavy oilfields, include differences in the relative and absolute concentrations of compounds such as n-alkanes, and isoprenoid alkanes which are relatively easily degraded components and typically decrease in concentration toward oil-water contacts. As discussed below, oil-water contacts are the primary site of oil biodegradation.

By contrast cyclic biomarker alkanes such as hopanes are more resistant to biodegradation and often increase in concentration toward the oil-water contact at low and intermediate levels of degradation. There are also, systematic molecular changes in the relative abundance of multiple components with very similar densities through the oil columns. This includes for example, decreasing isoprenoid alkane abundances relative to hopanes, or regular sterane abundances relative to diasteranes (Peters and Moldowan, 1993; Peters et al., 2005) which indicate the biodegradation process is driven by molecular selectivity rather than by gravitational processes related to compound density. Other examples include selective removal of individual aromatic hydrocarbon isomers of similar density toward the bottom of oil columns (e.g., alkylphenanthrenes, napththalenes, or dibenzothiophenes), which unambiguously indicate that biodegradation, not gravitational effects control oil composition variations in heavy oilfields.

While some degree of water washing may also have taken place penecontemporaneously (almost at the same time), with biodegradation, selective removal of specific alkylaromatic hydrocarbon or non-hydrocarbon isomers, with similar water solubilities (Taylor et al., 2001), suggests that biodegradation is dominant as the alteration process, and water washing is of little significance.

In this hypothesis and theory article we propose new ideas and explore emerging paradigms about the controlling factors that dictate in-reservoir petroleum biodegradation and the microbial ecology of heavy and other petroleum systems, and offer a perspective on the implications for energy recovery and carbon management in the future.

## Controls on in-reservoir petroleum biodegradation

Field observations typically record the lowest oil quality and the strongest biological and molecular evidence for hydrocarbon degradation at oil-water transition zones (OWTZ), suggesting that most petroleum degradation occurs at this interface (Head et al., 2003; Larter et al., 2003); where the biosphere meets the geosphere. This conclusion is sensible because at the oil-water contact, organisms find the water necessary for life along with electron donors and acceptors and carbon sources (oil) necessary to conserve energy and generate biomass. Essential nutrients, such as nitrogen and phosphorus are primarily derived from mineral buffering in the water leg (cf. Rogers et al., 1998; Head et al., 2003; Bennett et al., 2013).

Multiple and complex oil-water contacts may exist throughout a charging oil reservoir. However, the large vertical compositional gradients in oil columns of degraded oil reservoirs with the most degraded oils at the oil-water contact (Moldowan and McCaffrey, 1995; Horstad and Larter, 1997; Huang et al., 2003; Larter et al., 2003; Bennett et al., 2013) suggest the bulk of the biodegradation in most reservoirs is ultimately driven from the basal oil-water contact of the filling reservoir. Although petroleum biodegradation undoubtedly occurs in shallow carrier systems too, relative residence time in-reservoir, compared to in-carrier, is large, suggesting reservoir processes dominate alteration of oil.

The past decade has witnessed fundamental changes in our concept of in-reservoir petroleum biodegradation with a shift from a model that emphasized the central importance of aerobic oil degradation, with oxygen delivered to petroleum reservoirs in meteoric water (Palmer, 1993) to a model where in-reservoir crude oil biodegradation is driven by anaerobic processes. In reservoirs with low concentrations of sulfate, methanogenic degradation is a primary mechanism of petroleum biodegradation (Head et al., 2003; Larter et al., 2005; Jones et al., 2008). This conclusion was based on field measurements of metabolites characteristic of anaerobic hydrocarbon degradation (Aitken et al., 2004), comparative analysis of field-degraded oils with oils degraded under methanogenic or sulfate-reducing conditions in laboratory incubations, analysis of gas isotopes from the field and Rayleigh fractionation modeling (Jones et al., 2008). This conceptual shift has important implications for understanding the factors that control biodegradation *in situ*, and therefore, for establishing the likelihood that a prospect will be biodegraded pre-drill. Moreover, the mechanisms whereby petroleum hydrocarbons are degraded may dictate which diagnostic metabolites might act as signatures of different processes occurring in relation to prevailing reservoir conditions (e.g., low sulfate clastic systems vs. high sulfate carbonate systems). The following sections detail the factors affecting anaerobic and specifically methanogenic crude oil biodegradation.

### Oil properties

Here we evaluate the effects of different oil components on the methanogenic degradation of crude oil using both observations from field biodegraded oils and experimental approaches.

#### Do water soluble oil components retard methanogenic degradation of crude oil?

**Figure 1:**
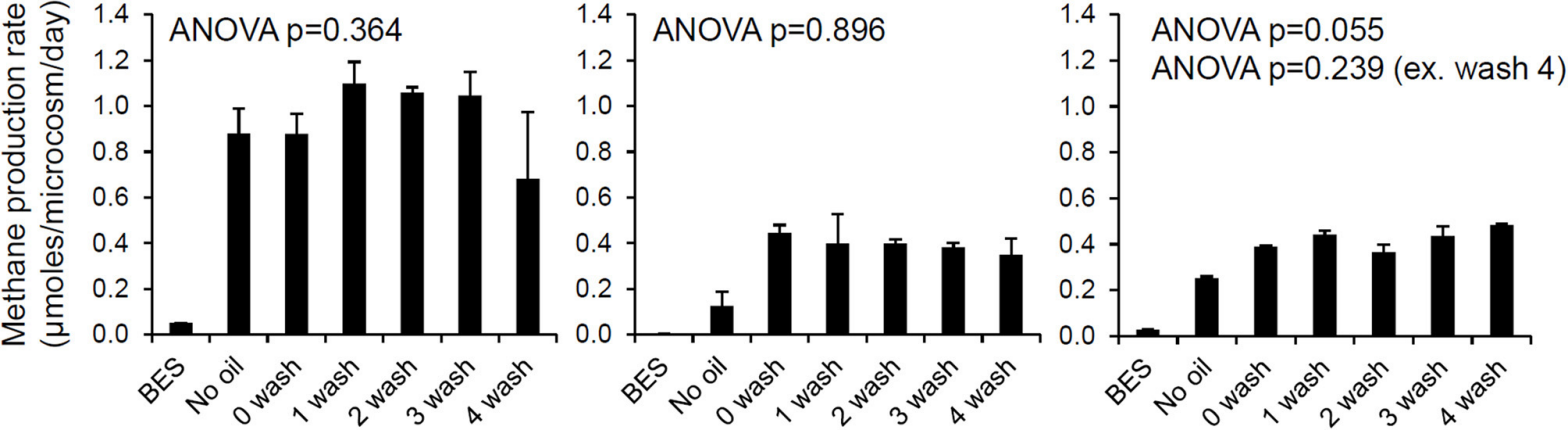
**The effect of oil and water-washed oil on rates of methanogenesis from H_2_/CO_2_ (left), acetate (middle), and methanol (right)**. Laboratory microcosms amended with oil which had been washed with brine to different degrees was evaluated. North Sea oil (50 g) was washed with 100 ml brine (1% w/v NaCl in deionized water) in a separating funnel from 1 to 4 times and the unwashed and washed oil (300 mg) was added to serum bottles containing a slurry of river sediment (River Tyne, UK) amended with each of the methanogenic substrates. Control incubations containing bromoethanesulfonic acid (BES), an inhibitor of methanogenesis were also prepared. Rates of methane production were measured by gas chromatography of headspace samples (E. Bowen, previously unpublished data).

It has been suggested that reservoir geometry has an important effect on the extent of biodegradation of crude oil in petroleum reservoirs (Larter et al., 2005, 2006). This has been interpreted principally in relation to the availability of water and supply of inorganic nutrients from the water leg, controlled by the area of the oil water contact in relation to the size of the reservoir and the size of the underlying aquifer. However, this could also be interpreted in terms of water washing with removal of inhibitory, water soluble components of the oil such as phenols. It has been suggested that petroleum reservoirs are biostatic (Sunde and Torsvik, 2005) i.e., biological activity is inhibited but the organisms present are not killed. Biostatic conditions in petroleum reservoirs have been explained in terms of toxic water soluble hydrocarbons or other components of the oil such as metals. It might also relate to toxic low molecular weight, volatile components of crude oil as inhibitors (see Section Do Volatile Hydrocarbons Influence Methanogenic Degradation of Crude Oil?). The suggestion that oil reservoirs are biostatic is based on the common observation that while oilfield waters often contain sulfate-reducing bacteria, electron donors, oxidants and nutrients, spontaneous H_2_S production is not observed until the oilfield water is degassed or diluted, when, H_2_S production subsequently takes place (Sunde and Torsvik, 2005). The biostatic nature of reservoir waters is paradoxical in the context of demonstrable in-reservoir oil biodegradation and therefore would imply a further control on oil biodegradation *in situ* which may relate to the activity of any associated aquifers. For instance reservoirs associated with an active aquifer where large volumes of water pass the oil water contact will be biodegraded, as toxic, water soluble oil components would be washed from the oil. This hypothesis would also be consistent with the lack of oil biodegradation in reservoirs with an under seal with oil completely filling the reservoir compartment down to the under seal. At field scale, it is not clear how the effects of removing or diluting toxic water soluble compounds could be distinguished from enhanced delivery of nutrients by a more active aquifer. Interestingly, new results presented here show that water washing of oil has little effect on rates of methanogenesis in laboratory incubations (Figure 1). From these results it seems unlikely that readily water-extractable toxic polar compounds in oil inhibit methanogenic crude oil biodegradation or explain the prevalence of methanogenic CO_2_ reduction sometimes seen during crude oil biodegradation and described as MADCOR (Methanogenic Alkane Degradation Dominated by CO_2_ Reduction; Jones et al., 2008). A possible explanation of MADCOR is preferential inhibition of acetoclastic methanogens (Warren et al., 2003, 2004; Westerholm et al., 2011). Interestingly Warren et al. (2003, 2004) noted that aqueous extracts of creosote were inhibitory to acetoclastic methanogenesis. The amounts of oil used in the laboratory experiments reported here were small (Figure 1). In general in many petroleum systems, oil contacts relatively small volumes of water compared with the volume of the reservoir, and this also suggests that water soluble inhibitors are in reality not a primary factor in driving MADCOR.

#### Do volatile hydrocarbons influence methanogenic degradation of crude oil?

Several studies indicate that low molecular weight hydrocarbons inhibit the aerobic biodegradation of crude oil (Westlake et al., 1974; Atlas, 1975; Walker et al., 1976; Wang et al., 1998). This may explain why aerobic biodegradation of spilled oil is not observed until volatilization has removed low molecular weight alkanes (Atlas and Bartha, 1972b; Whittle et al., 1982). Moreover there is evidence that some, but not all anaerobic bacteria are inhibited by short chain alkanes (<C9; Rodriguez-Martinez et al., 2008). This may also explain the observation of biostatic conditions that prevent sulfate reduction in petroleum reservoirs.

Interestingly a comparison of methanogenic oil-degrading microcosms provided with a North Sea oil containing volatile aromatic and aliphatic hydrocarbons with the same oil from which the volatile components (alkanes < C11 and monoaromatic hydrocarbons) were removed (by incubation of the oil at 25°C for 48 h) demonstrated that methanogenic oil degradation occurred after a much shorter time period, and at a much faster rate in microcosms lacking the volatile hydrocarbons (Sherry et al., 2014). Nevertheless oil degradation was not completely inhibited in the presence of the volatile hydrocarbons and thus may have an important role to play in modulating the rate of in-reservoir crude oil biodegradation.

### Environmental constraints on anaerobic crude oil biodegradation

A number of environmental factors are known to affect microbial activity, these include temperature, pH, salinity, water activity, radiation and availability of resources such as carbon and energy sources, electron acceptors and inorganic nutrients. For in-reservoir crude oil biodegradation the most important of these are temperature, salinity, and inorganic nutrient availability.

#### Temperature

Connan (1984) first suggested that in-reservoir oil biodegradation ceased above a reservoir temperature of around 80°C. However, non-degraded reservoirs are also found at lower temperatures and an explanation for their occurrence came with the development of the palaeopasteurization hypothesis, developed at the turn of the twentieth century (Wilhelms et al., 2001). The palaeopasteurization hypothesis, formulated based on field data, indicated that the upper thermal limit for hydrocarbon-degrading microbial life in petroleum reservoirs was 80–90°C and that once a reservoir had been heated to temperatures within this range, it was not re-colonized by hydrocarbon degrading microorganisms, even if the reservoir was subsequently uplifted to shallower cooler depths (Wilhelms et al., 2001). Subsequently a systematic relationship between biodegradation, reservoir depth, and burial temperature was observed in the Western Canada Sedimentary Basin (Adams et al., 2006). Thus, temperature is considered a primary control on the occurrence of biodegraded petroleum reservoirs.

From the literature it appears that thermophilic hydrocarbon-degrading microorganisms are rare. One moderately thermophilic alkane-degrading sulfate reducing bacterium (*Desulfothermus naphthae* strain TD3), with optimum activity at 60°C has been isolated from Guaymas Basin sediments (Rueter et al., 1994), which degraded low molecular weight alkanes. It is only recently that hydrocarbon degradation at relatively high temperatures has been reported in laboratory incubations inoculated from petroleum reservoirs under both sulfate-reducing and methanogenic conditions (Gieg et al., 2010; Mbadinga et al., 2012; Zhou et al., 2012). These findings are supported by studies of pure and enrichment cultures that demonstrate the hydrocarbon-degrading ability of a range of thermophilic and hyperthermophilic anaerobic bacteria and archaea. Chen and Taylor (1997) reported the degradation of some BTEX hydrocarbons at 50°C by a sulfate-reducing enrichment culture. Additionally, propane degradation at 60°C occurred in a sulfate-reducing enrichment dominated by a spore-former related to *Desulfotomaculum* (Kniemeyer et al., 2007) and short chain alkane degradation linked to sulfate reduction occurs in hydrothermal sediments at temperatures up to 75°C (Adams et al., 2013b).

Hyperthermophilic archaea are also now known to degrade hydrocarbons. Isolates of the sulfate-reducing archaeon *Archaeoglobus fulgidus* have been obtained from hydrothermal systems and petroleum reservoirs with some indications that isolates from different habitats are very similar at the genomic level (Stetter et al., 1993). Early evidence showing that *Archaeoglobus* could grow on crude oil indicated that it was most likely fatty acids in the oil that served as growth substrates (Stetter et al., 1993). However, it was subsequently demonstrated that the type strain of *A. fulgidus* (strain VC 16) could grow on alkenes (Khelifi et al., 2010) and long chain alkanes (Khelifi et al., 2014) at 70°C. Another member of the *Archaeoglobales, Ferroglobus placidus*, degrades benzene with ferric iron as an electron acceptor at 85°C. This is the current upper limit for anaerobic hydrocarbon degradation by a pure microbial culture (Holmes et al., 2011). There is also some evidence to suggest that *Thermococcus sibiricus* may have the capacity to degrade alkanes (Mardanov et al., 2009).

While these studies bring hydrocarbon degradation into the realm of the hyperthermophiles, data remain consistent with the upper temperature limit for hydrocarbon-degrading life in the deep petroleum biosphere inferred from the palaeopasteurization hypothesis (Wilhelms et al., 2001). There are few reports of deep subsurface hyperthermophiles that have been isolated at temperatures greater than 90°C when oilfield waters have been used as an inoculum (Grassia et al., 1996) and, interestingly, methanogenesis and sulfate reduction could be measured in produced waters from Californian petroleum reservoirs only at temperatures between 70 and 83°C and not at higher temperatures, even when the temperature of the reservoir from which the samples came, was up to 120°C (Orphan et al., 2003). It is also of interest that the organisms implicated in hydrocarbon degradation around the field-derived palaeopasteurization threshold, use electron acceptors offering relatively high energy yields and there have to date been no reports of methanogenic oil degradation at such high temperatures. It is also of note in this regard, that *A. fulgidus* can degrade alkenes with either sulfate or thiosulfate as an electron acceptor (Khelifi et al., 2010), while degradation of alkanes was only reported with thiosulfate, and not the less energetically favorable sulfate, as an electron acceptor (Khelifi et al., 2014).

**Figure 2:**
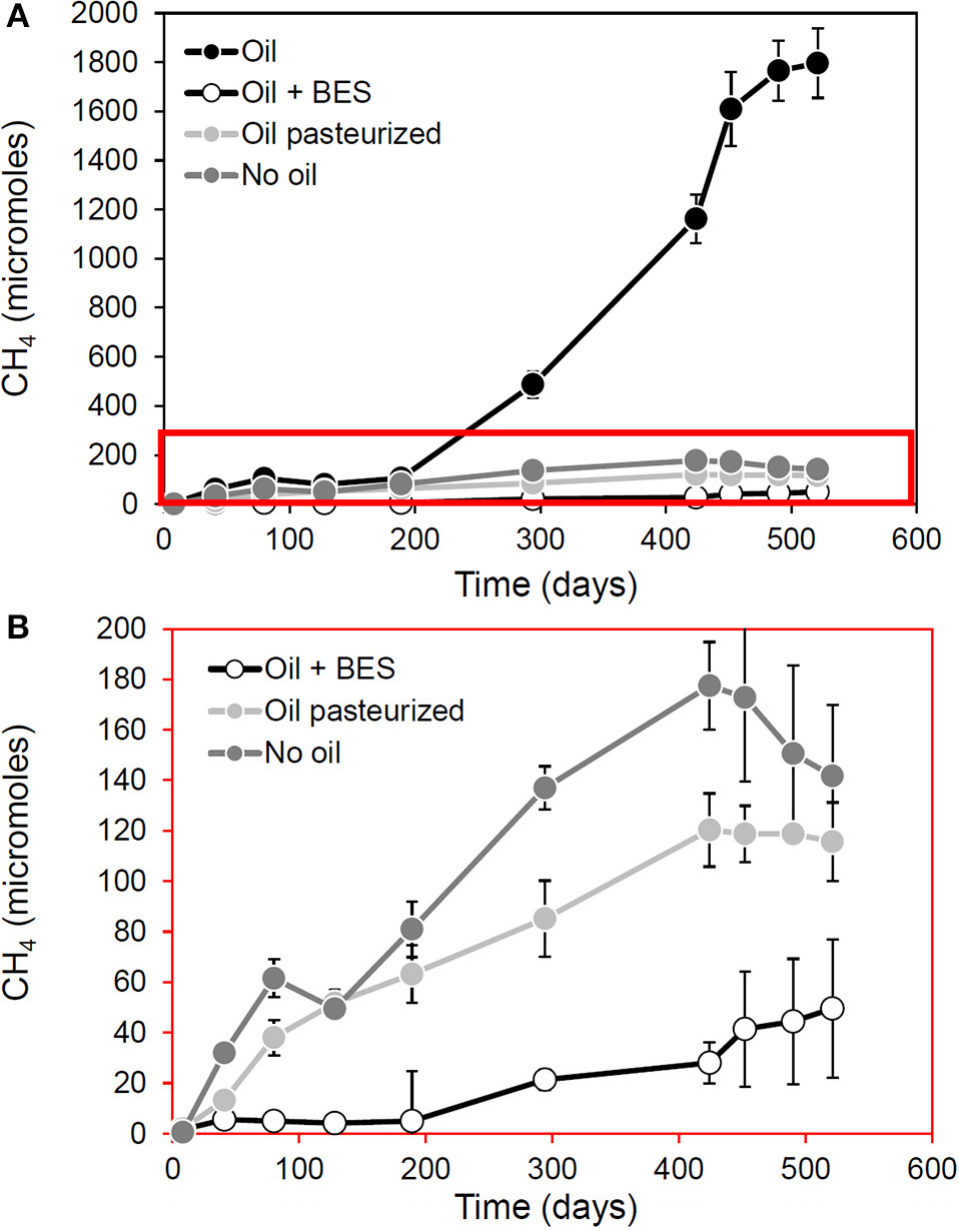
**Effect of pasteurization in methanogenesis and methanogenic oil biodegradation in laboratory microcosms**. Panel **(B)** is a blow up of the red box in **(A)**. This demonstrates that following pasteurization (90°C for 2 h) methanogenesis attributable to oil biodegradation (“Oil” in **A**) is reduced. The data from pasteurized control microcosms amended with oil suggest that methanogens remain active, even after pasteurization has removed hydrocarbon degrading activity. This is evident from the fact that pasteurization does not completely inhibit methanogenesis but brings methane production levels in line with that seen in background control incubations (No oil) to which no oil was added suggesting that the capacity for conversion of alkanes to methane has been removed by pasteurization, but not the ability to generate methane from indigenous organic carbon in the sediments (A. Rowan, previously unpublished data).

Observations from laboratory and field measurements are also informative. Re-examination of data from our own laboratory shows that methanogensis from oil degradation (but not from indigenous organic matter) is inhibited by pasteurization (Figure 2). This suggests hydrocarbon fermenting bacteria which provide methanogens with hydrogen, carbon dioxide and acetate are more heat-sensitive than the methanogens themselves since pasteurized sediments containing crude oil continue to produce methane but only at levels equivalent to control incubations to which no oil has been added (Figure 2).

**Figure 3:**
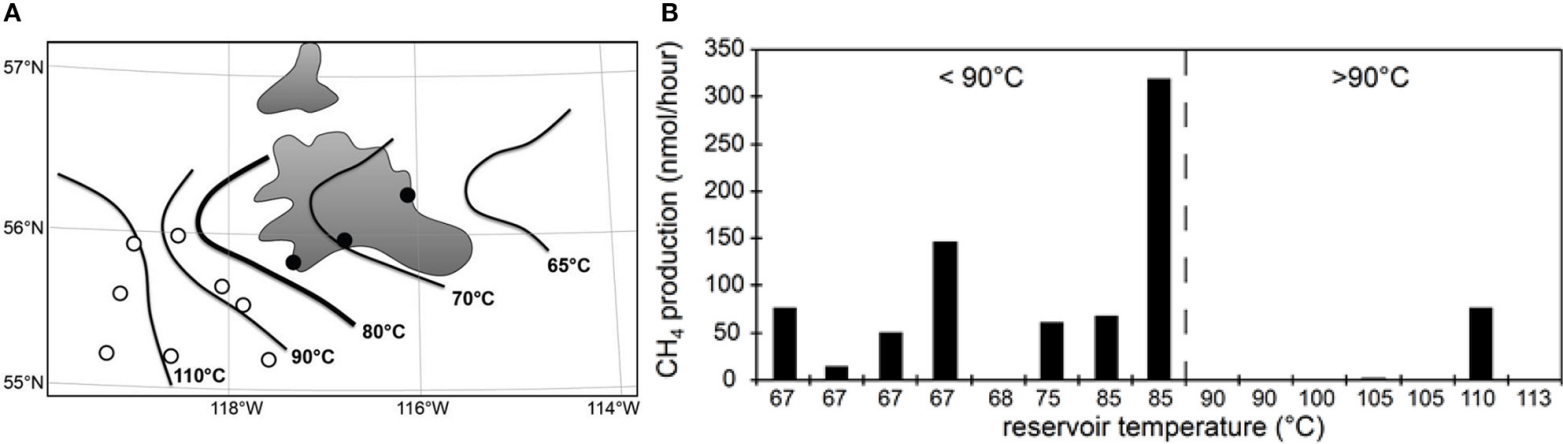
**Oil production wells (circles) near the Peace River tar sands (gray shaded areas) in Alberta (A)**. Isotherms indicate *in situ* temperatures of crude oil deposits. Consistent with palaeopasteurization, we recently recovered microbial intact polar lipids in produced water samples from wells east of the 80°C isotherm (●) but not from more western reservoirs (○) that are above 80°C (Oldenburg et al., 2009). Panel **(B)** shows that microbial activity (methanogenesis from yeast extract) was readily stimulated in samples from eastern fields lower than 80–90°C *in situ* (dashed line) but not from deeper, hotter fields to the west (N. Gray, unpublished data).

A lower thermal maximum for microbial life in petroleum systems is also evident from measurements of methanogenesis from complex organic matter (yeast extract) in water samples from petroleum reservoirs in the Western Canada Sedimentary Basin that span a temperature gradient from 67 to 113°C (Figure 3). Methanogenesis could be measured in almost all samples from reservoirs with *in situ* temperatures below 90°C but only in one reservoir above this temperature (previously unpublished data, Figure 3). Furthermore, it was possible to recover intact bacterial polar lipids (IPL) only from lower temperature reservoirs (Oldenburg et al., 2009). A number of lines of evidence therefore suggest that in petroleum reservoirs the maximum temperature for biological activity is below that observed for cultivated organisms isolated from high temperature hydrothermal systems (121–122°C; Kashefi and Lovley, 2003; Takai et al., 2008). Nevertheless, intact prokaryotic cells have been identified in deep sediments where temperature exceeds 80–90°C. Mud volcano breccia inferred to emanate from depths where the temperature is estimated to be up to 160°C (Parkes et al., 2000), sediments from the Newfoundland Margin [1626 mbsf, 111 My and 60–100°C (Roussel et al., 2008)] and deep coal seams in New Zealand (estimated maximum temperature 80–90°C; Fry et al., 2009) have all been shown to harbor large numbers of prokaryotic cells, though their activity, and specifically their hydrocarbon degradation potential, at these temperatures is unknown.

One reason for a modulated maximum thermal limit for life in some deep subsurface sediments may be that the rates of metabolism that can be sustained in a metabolically constrained system do not allow for the regeneration of labile molecules involved in conservation of energy (e.g., ATP and NADH) sufficiently quickly to support the maintenance energy requirements of cells (Wilhelms et al., 2001). It has been shown that cells with low metabolic activity have reduced tolerance to environmental extremes (Lloyd et al., 2005). This may be exacerbated by additional energy requirements to maintain cell integrity in a chemically demanding environment such as crude oil. There are many other extremes found in deep subsurface sediments that may also conspire to limit the tolerances of microbial life and it is known that interactions between different environmental factors can act to increase or decrease an organism's sensitivity to other environmental stressors (Edgcomb et al., 2004).

#### Salinity

It is well known that salinity affects microbial activity and that petroleum reservoir formation waters can vary from freshwater, to many times seawater salinity. Reservoir water salinity will therefore affect the occurrence of biodegraded oils. Furthermore, it is likely that there will be an interaction between salinity and other environmental factors such as temperature, and for example, the temperature required for palaeopasteurisation of a reservoir may be lower if the salinity is elevated. Although there is no systematic analysis of salinity-biodegradation relationships in oil fields, there is anecdotal evidence that some low temperature, non-uplifted, non-biodegraded reservoirs are associated with high salinity aquifers. We therefore propose that “Palaeopickling” (cf. palaeopasteurisation) may be a mechanism whereby biodegradation of crude oil is prevented, providing a further factor that should be considered for pre-drill prediction of biodegradation.

***Salinity tolerances and mechanisms of osmoadaptation in microorganisms***. Microorganisms are known that can grow over the full salinity range from almost pure water to saturated brines and particular organisms are adapted to particular ranges of salinity (DasSarma and Arora, 2002). To counteract the effect of osmotic efflux of water in high solute environments microorganisms have evolved two main mechanisms;

- accumulation of ions (typically K^+^ and Cl^−^ ions) to high intracellular concentrations (the “salt in” strategy) and
- intracellular synthesis of organic compounds known as compatible solutes (so called because they are compatible with the activity of enzymes).

Both the “salt in” and solute synthesis strategies require considerable energy expenditure with the synthesis of compatible solutes being energetically more demanding than accumulation of ions. This energy cost must be deducted from the energy yield from primary metabolism. This typically means that the upper salinity limit for growth and activity is lower for organisms that live by harnessing reactions that have low energy yields (Oren, 2010). Although, low energy yield *per se* does not preclude activity at higher salinity, the rate at which energy can be supplied does. Given a finite metabolic activity, the point at which energy can no longer be supplied at a sufficient rate to service cell maintenance, dictates when a microbial process will cease.

**Figure 4:**
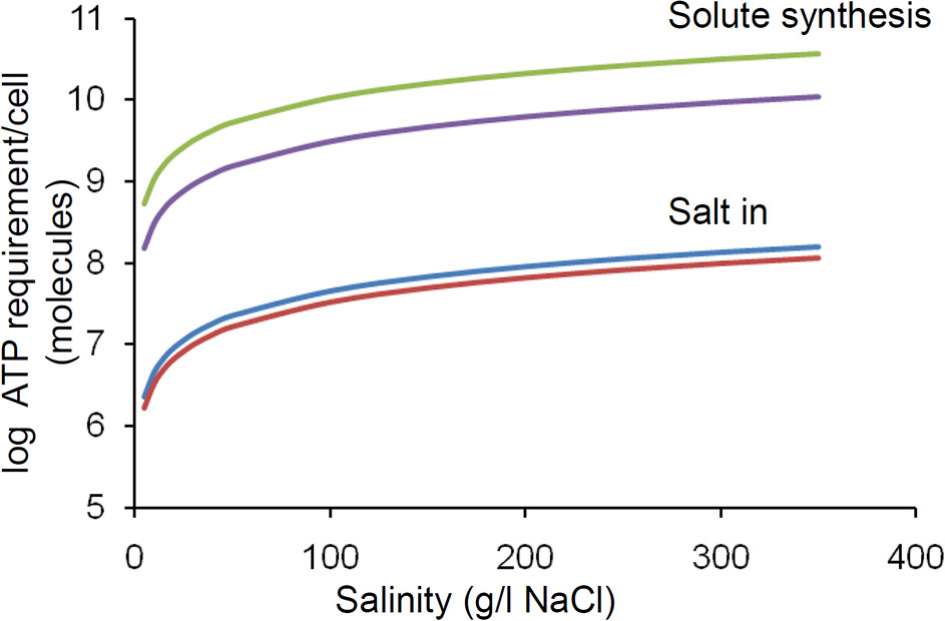
**Effect of increasing salinity on energy (ATP) requirement/cell for osmoregulation**. The upper and lower lines in each pair of lines relates to the upper and lower values quoted by Oren (1999). Calculations were based upon the following assumptions; Intracellular solute concentration is regulated to match the extracellular salinity; For organic solutes 2 molecules are required per salt molecule (1 molecule of salt produces 2 ions); Cells are cocci (spheres) of 0.5 microns in diameter. Note log scale, thus there is a linear increase in energy demand with increasing salinity.

Synthesis of compatible solutes takes from 20 to >100 moles of adenosine triphosphate (ATP) per mole of compatible solute depending on the specific molecule (Oren, 1999). In principle the internal concentration of the compatible solute must be twice the external salt concentration (NaCl dissociates into 2 ions). In practice this value is lower due to the osmolarity contribution from normal intracellular solutes including proteins but still represents a considerable metabolic burden on a cell which is linearly proportional to the salinity. We have estimated that the ATP requirement per cell increases by an order of magnitude between 0 and 400 g/L salt concentration (Figure 4). The salt in strategy is less energetically expensive, but still exerts a similar proportional increase in burden over this range (Figure 4).

Salinity effects on methanogenesis, the terminal process in methanogenic oil biodegradation depends on the specific pathway utilized, with tolerance increasing with increasing substrate energy yield. Typically, acetoclastic methanogenesis yields least energy and has a relatively low upper salinity limit (Oren, 2010). Methanogenesis from H_2_ + CO_2_ has a higher salinity tolerance but has never been shown above 175 gl^−1^ salt (Zhilina et al., 2013). At higher salt concentrations, biogenic methane comes from disproportionation of methylated amines which yield more energy than other methanogenic substrates. Of course the specifics will be dictated by *in situ* conditions such as acetate or hydrogen concentration which will affect metabolic rates and the overall energy available in the system.

***Salinity effects in the context of methanogenesis in petroleum reservoirs***. It has been noted that the frequency of isolation of microbial cultures from petroleum reservoirs decreases above a salinity of ca. 100 g/l (Grassia et al., 1996; Röling et al., 2003). Furthermore, methanogenesis from reduction of CO_2_ with H_2_, the most prevalent methanogenic process seen in petroleum reservoirs (Head et al., 2010), has never been detected in natural environments at salt concentrations exceeding 100 g/l. This is consistent with the observation of methanogenesis from CO_2_ with H_2_only at salinities up to 90 g/l in a high temperature, saline North Sea oil and gas reservoir, where the *in situ* salinity was 90 g/l (Gray et al., 2009).

However, the cultured H_2_-oxidizing CO_2_ reducing methanogen, *Methanocalculus halotolerans*, was, isolated from an oil well and grows at salt concentrations up to 120 g/l NaCl and at temperatures up to 45°C (Olivier et al., 1998). It is likely that the discrepancy between the salt tolerance of methanogenesis by *Methanocalculus halotolerans* and *in situ* methanogenesis rates is the fact that the maximum growth temperature of *Methanocalculus halotolerans* is somewhat lower than the *in situ* reservoir temperature and it is possible that at higher temperatures the salinity tolerance of this methanogen may be lower. This is the first circumstantial evidence that there may be an interaction between salinity and temperature on microorganism activity and the possibility that palaeopickling of low temperature reservoirs with very saline formation waters may prevent biodegradation.

**Figure 5:**
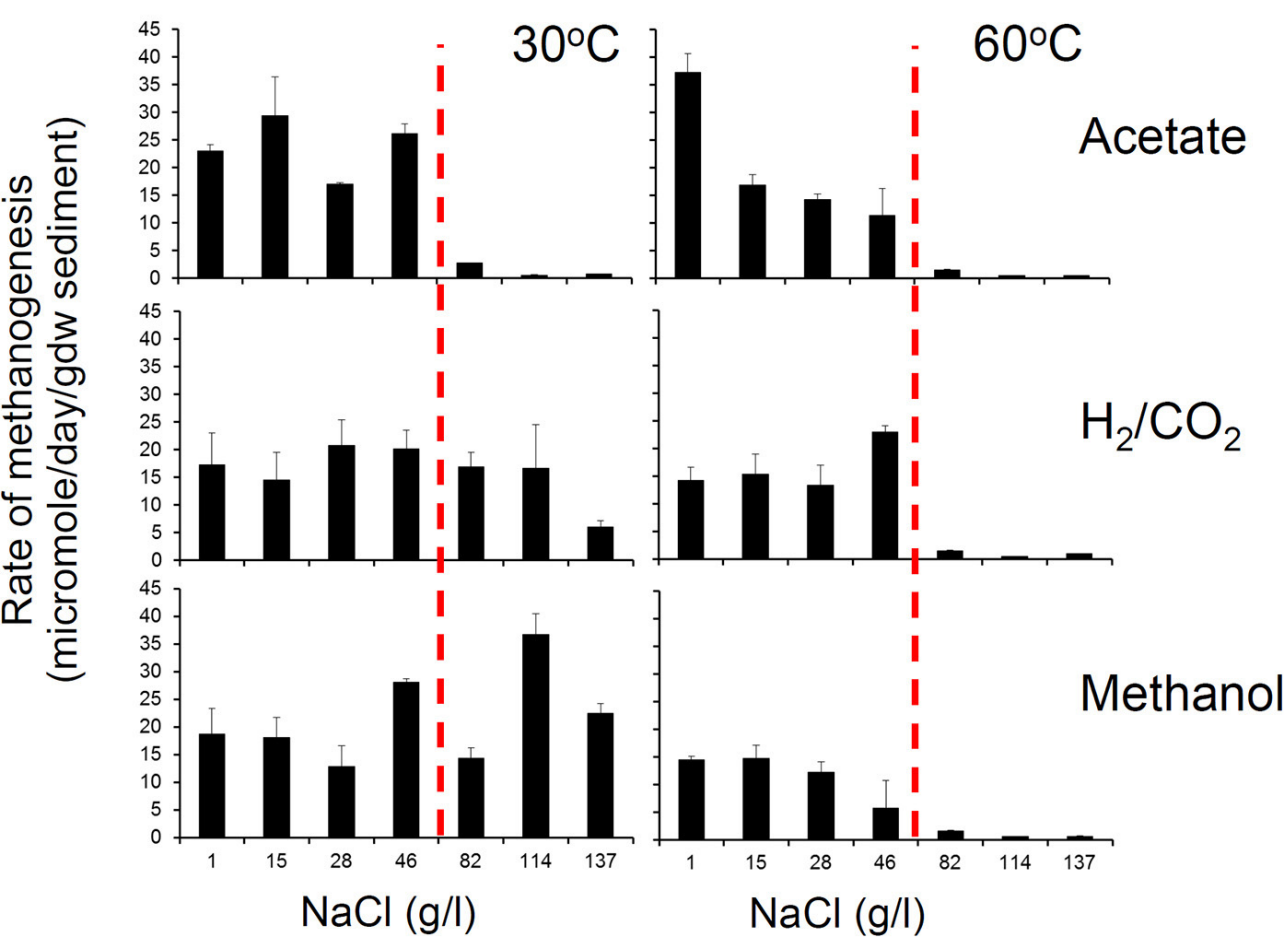
**Methanogenesis from acetate, hydrogen and CO_2_ and methanol in anoxic incubations of River Tyne sediments incubated at 30°C (left panel) and 60°C (right panel) over a range of salt concentrations from 1 to 137 g/l**. The maximum salt tolerance for methanogenesis is lower at higher temperature indicating an interaction between salinity and temperature (H. Coombs, previously unpublished data). At 60°C methanogenic activity is inhibited above 46 g/l indicated by the dashed vertical red line.

We have conducted a systematic analysis of the effect of salinity on rates of methanogenesis and report the results here. This was done using river sediment microcosms to which different levels of NaCl were added to give a range from 1 to 137 g/l NaCl and these were amended with a range of methanogenic substrates. Two sets of incubations were conducted, one at 30°C and one at 60°C, and rates of methane production were measured. These experiments clearly demonstrated that there was an interaction between temperature and salinity with the salinity tolerance of methanogenesis from all methanogenic substrates being greater at 30°C than at 60°C. At 30°C, the maximum salt concentration permissive for methanogenesis from acetate, CO_2_ and H_2_, and methanol was 46, 114, and 137 g/l NaCl respectively (Figure 5), confirming the observations of Oren (2010). However, at 60°C methanogenesis from all substrates ceased above 46 g/l NaCl (Figure 5).

Clearly the terminal processes in crude oil degradation under methanogenic conditions show an interaction between salinity and temperature that may affect the occurrence of in-reservoir oil biodegradation. How might this translate into effects on biodegradation of oil? As indicated above, the energetic cost of osmoadaptation is high and proportional to the salt concentration and thus processes that yield less energy are likely to be compromised by high salinity conditions (Oren, 2010). These are the very processes that are central to methanogenic oil biodegradation. In addition to acetoclastic methanogenesis, syntrophic proton reducers i.e., primary alkane fermenters and syntrophic acetate oxidizers, yield small amounts of energy and have relatively limited salinity tolerance. These processes coupled to methanogenesis are the principal processes in methanogenic degradation of crude oil alkanes (Gieg et al., 2008; Jones et al., 2008). Taken together these data and observations suggest that palaeopickling may be important for preserving oil in low temperature petroleum reservoirs and that the palaeopasteurization temperature required to preserve oil may be lower in high salinity systems, potentially providing a further approach to inform pre-drill prediction of biodegradation. We predict that at higher salinity the palaeopasteurization temperature is likely to be reduced from the maximum field observed value of 80°C. However, the precise relationship between salinity, temperature and in-reservoir oil preservation is currently unknown. A systematic analysis and calibration against field data from petroleum reservoirs is therefore much needed.

#### Nutrient availability

A primary control on the degradation of crude oil in *aerobic* environments is nutrient availability (Atlas and Bartha, 1972a; Head et al., 2006). Degradation relies on the growth of hydrocarbon-degrading organisms and hydrocarbon conversion to biomass for growth requires additional nitrogen and phosphorus. These nutrients make up a significant proportion of biomass and are typically around 15% N and 1% P on a dry weight basis (Redfield, 1934, 1958) though this does vary (e.g., Fagerbakke et al., 1996; Gunderson et al., 2002; Vrede et al., 2002). Thus, to convert hydrocarbons to biomass, supplementary N and P are required. This is one of the reasons that nutrient availability is also considered an important control on in reservoir oil biodegradation. However, anaerobes typically have lower biomass yields than aerobes and growth yields of methanogenic hydrocarbon degrading consortia are in the range of a 2–10% compared to the 50% growth yield expected for aerobic heterotrophs (Gray et al., 2011). Thus, in methanogenic oil degrading systems such as biodegrading petroleum reservoirs proportionately less N and P are required to permit degradation of a given mass of hydrocarbon coupled to growth.

There are few data on inorganic nitrogen concentrations in deep biosphere sediments. Relatively shallow (219 m) and young sediments (4,60,000 y) from the northwestern Pacific Ocean contained ammonium concentrations of 15 mM and based on stable isotope tracer studies with ^15^N-labeled ammonium it was concluded that growth and activity were energy- and not inorganic nutrient-limited (Morono et al., 2011). Typically deep subsurface sediments are considered carbon and energy limited as organic carbon quality and quantity decrease with depth and microbial biomass correlates with organic carbon content (D'Hondt et al., 2009). Petroleum reservoirs are different as they contain large quantities of potential electron donor. Deep groundwater from petroleum reservoirs may contain substantial amounts of ammonium [up to 1000 mg/l (55 mM); Manning and Hutcheon, 2004]. Intriguingly, in a study of oil sand reservoirs, conservative estimates of formation water N and P indicated that cell counts, on the order of those observed in formation waters (ca 10^5^–10^6^ per gram), can be supported without removing additional nutrients from the oil or surrounding rock thus N and P availability does not appear to constrain the degree of oil biodegradation in this system.

While inorganic nitrogen is the likely immediate source of nutrients for the in-reservoir oil biodegradation system, petroleum contains considerable amounts of organic nitrogen. Total nitrogen content of crude oils range between 0.01 and 0.9% by weight (Ball et al., 1951). However, Richter et al. (1952), suggested that most of the organic nitrogen is present in the high molecular weight fraction of crude oils. The low molecular weight fraction of crude oil, in terms of its nitrogen compounds is dominated by alkylcarbazoles, and these are biodegraded at degradation levels above Peters and Moldowan level 4, and might supply nitrogen to microbial communities resident in petroleum reservoirs (Oldenburg et al., 2006). Clearly, alkylcarbazole-derived nitrogen is not used during the early stages of biodegradation when alkane removal is the primary process occurring. Thus, oil-derived nitrogen may not be quantitatively critical at the times when the highest rates of hydrocarbon removal occur.

### Thermodynamic drivers

The accumulation of metabolic intermediates and end products of oil degradation may exert a thermodynamic control on in-reservoir crude oil biodegradation linked to methanogenesis.

#### Inhibition by accumulation of metabolic intermediates (H_2_)

**Figure 6:**
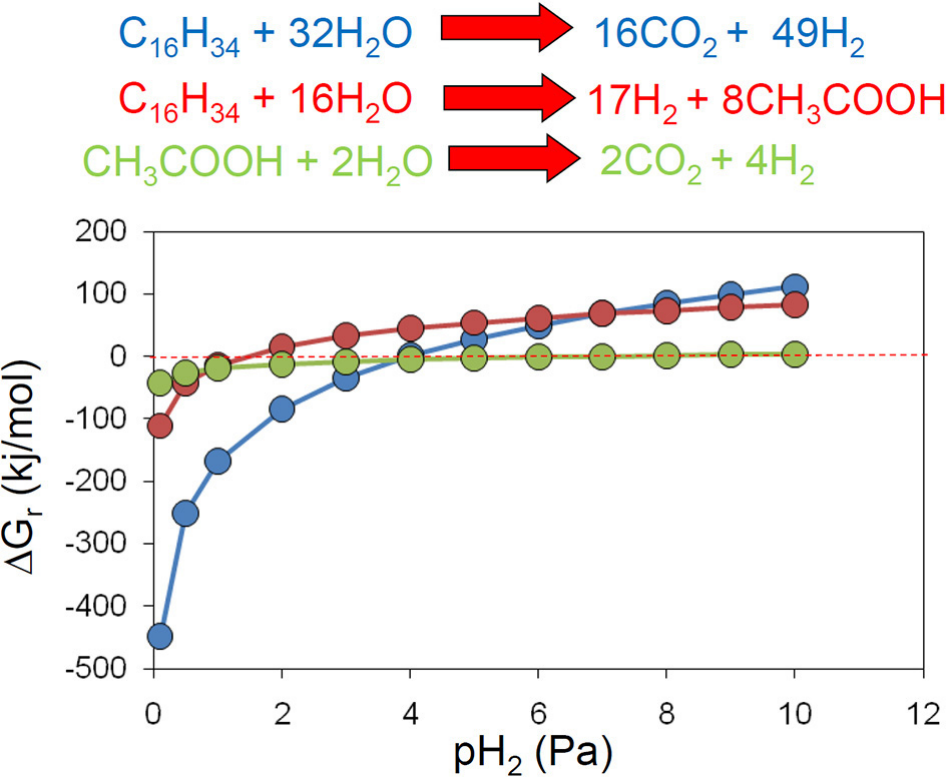
**The effect of hydrogen partial pressure on the free energy yield from oxidation of hexadecane to hydrogen and CO_2_ (blue), fermentation of hexadecane to hydrogen and acetate (red) and syntrophic acetate oxidation (green)**.

Methanogenic alkane degradation may proceed via several routes that can involve complete oxidation to hydrogen and carbon dioxide, fermentation to acetate and hydrogen and syntrophic acetate oxidation (Dolfing et al., 2008). Hydrogen is an important intermediate in these processes (Dolfing et al., 2008) and an examination of the effect of hydrogen partial pressure on the theoretical Gibb's free energy yield of each of these processes confirms that for the conversion of alkanes to methanogenic substrates a very low hydrogen partial pressure (less than 4 Pa/ 4 × 10^−5^ atm) is required if these processes are to remain thermodynamically feasible (Figure 6).

These low levels of hydrogen are typically maintained by methanogens that efficiently consume hydrogen produced by fermentation reactions, however if their activity is reduced due to inhibition by some environmental factor such as pH or presence of inhibitory compounds, then partial pressure of hydrogen can rise leading to a cessation of primary fermentation of the alkanes. Clearly then accumulation of metabolic intermediates such as hydrogen potentially has a profound effect on methanogenic crude oil biodegradation.

#### Inhibition by accumulation of metabolic end-products (CH_4_ and CO_2_)

**Figure 7:**
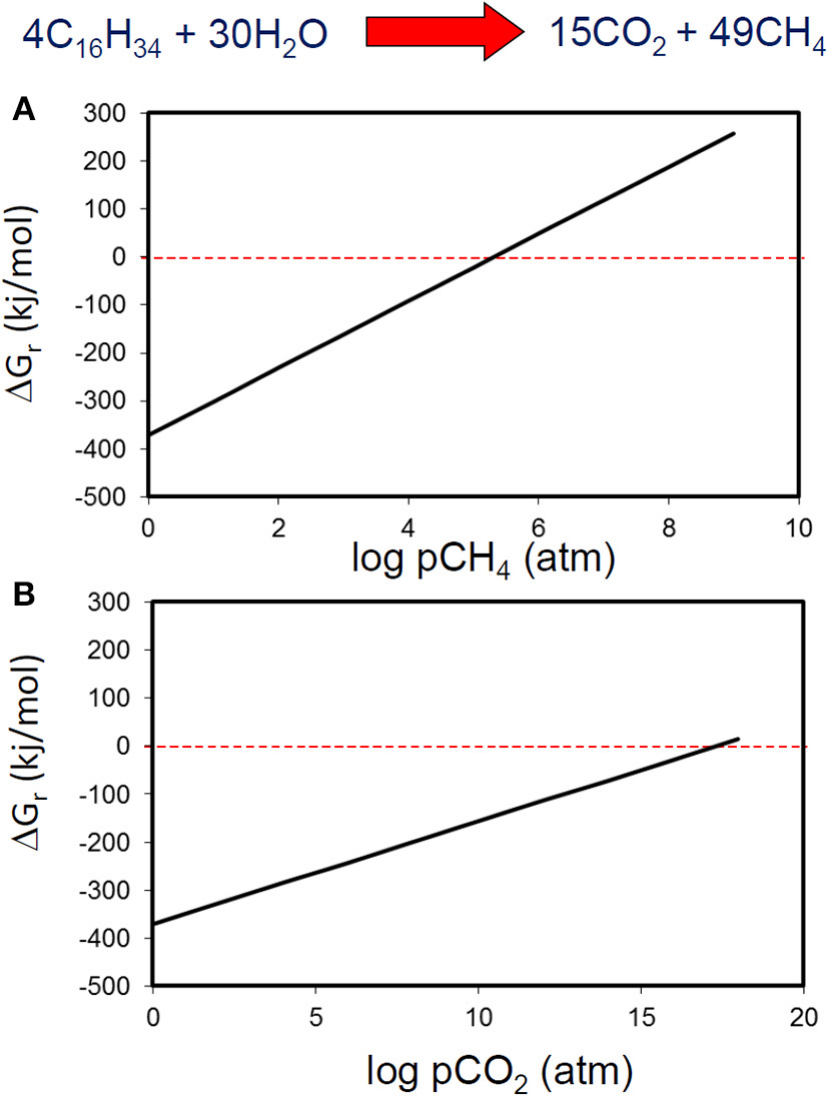
**Free energy yield from methanogenic hexadecane degradation at a range of methane (A) and carbon dioxide (B) partial pressures**.

It is relatively well known from studies of methanogenic systems that accumulation of metabolic intermediates such as hydrogen leads to thermodynamic inhibition of biodegradation of many compounds. However, it is usually assumed that metabolic end products have little effect on the thermodynamics of methanogenic systems. Using standard conditions except for methane concentration, we have calculated that methanogenic hexadecane degradation remains thermodynamically feasible even at methane concentration greater than 10^4^ atmospheres (Figure 7). Carbon dioxide concentrations are more permissive and the free energy change remains negative up to CO_2_ concentrations of 10^17^ atmospheres (Figure 7). These concentrations far exceed those likely to be attained in biodegrading petroleum systems. If methane and CO_2_ concentrations are considered together and a “window of opportunity” defined, where methanogenic alkane degradation is thermodynamically favorable (Dolfing et al., 2008) the window of opportunity is huge.

**Figure 8:**
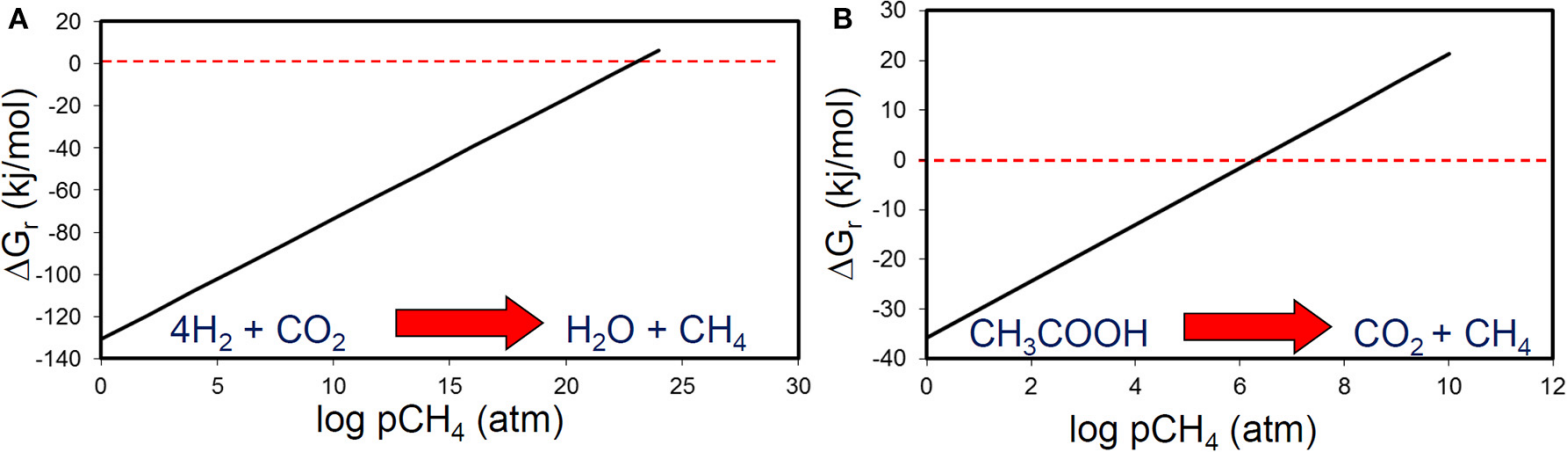
**The effect of methane partial pressure on the thermodynamic feasibility of (A) methanogenic CO_2_ reduction and (B) acetoclastic methanogenesis**.

**Figure 9:**
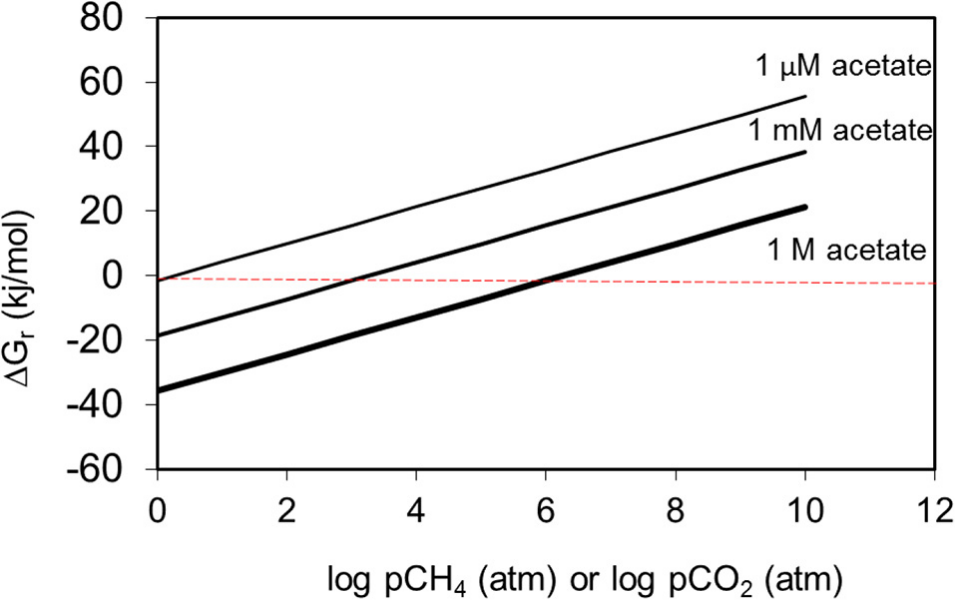
**The effect of methane or CO_2_ partial pressure on the thermodynamic feasibility of acetoclastic methanogenesis at different acetate concentrations**. At micromolar levels of acetate, acetoclastic methanogenesis becomes thermodynamically unfavourable at modest levels of methane or CO_2_.

Methane and CO_2_ partial pressures likewise, are unlikely to have a major impact on the thermodynamic feasibility of the main methanogenic pathways relevant for crude oil alkane degradation. For example, the free energy change of methanogenesis from CO_2_ reduction and acetoclastic methanogensis remain negative up to CH_4_ concentrations of 10^24^ and 10^6^ atmospheres respectively (Figure 8). It should, however, be noted that these calculations were conducted under standard conditions with all reactants and products at 1 M concentration (except methane). Interestingly, in the specific case of acetoclastic methanogenesis the effect of end product concentration is profound. At acetate concentrations that are realistic for petroleum reservoirs (micromolar levels), acetoclastic methanogenesis becomes thermodynamically unfavorable at rather modest methane and CO_2_ concentrations (a few atmospheres; Figure 9). This is possibly a further reason that under some circumstances the MADCOR process of hydrocarbon degradation is channeled through syntrophic acetate oxidation coupled with methanogenic CO_2_ reduction, rather than by direct acetoclastic methanogenesis (Jones et al., 2008). A further interesting consequence of increasing CO_2_ partial pressure from geological CO_2_ sequestration technologies is the observed shift in carbon flow from syntrophic acetate oxidation coupled with methanogenic CO_2_ reduction, to acetoclastic methanogenesis (Mayumi et al., 2013).

## Rates and fluxes—from fundamental science to oil field applications

Biological and biogeochemical inferences about subsurface petroleum biodegradation, have real-world applications. “Kinetic models,” of subsurface petroleum biodegradation are now routinely applied in basin modeling studies to assess oil properties prior to drilling expensive exploration or production wells. Based on our assessments of temperature controlled biodegradation rates and zero order biodegradation fluxes (net rates of hydrocarbon destruction, per square meter of oil water contact area within the reservoir), and the development of compositional biodegradation models for crude oil (Larter et al., 2003, 2006), several groups have now developed biodegradation models which consider effects on oil composition (Blumenstein et al., 2008; Haeseler et al., 2010). Larter et al. (2006), calibrated compound class specific or bulk oil degradation flux vs. reservoir temperature profiles using measured compositional gradients in actual oilfields and also used Monte Carlo simulations of large oil data sets from individual basins. The subsurface reservoir biodegradation fluxes (the rate at which petroleum compounds are destroyed in units of kilograms of “hydrocarbons,” per square meter of oil water contact area per year), for fresh petroleum in clastic e.g., sandstone, reservoirs are on the order of 10^−4^ kg/m^2^/year at 40°C decreasing with increasing reservoir temperature to a value close to zero, around 80°C. At very low reservoir temperatures (circa 10–20°C), such as seen in the near surface Canadian oil sands, the net degradation fluxes are much less than maximum values. Of course these rates are subject to the other controls e.g., toxicity, nutrients and salinity discussed above.

The degradation flux concept is easily integrated into basin modeling software (which simulates the geological development of basins, the maturation of source rocks, migration of oil and subsequent alteration processes of the oil within the reservoir) as it directly relates total degradation flux to evolving oil water contact area as oilfields develop. The biodegradation flux approach of Larter et al. (2006) enhanced by the incorporation of water leg size and oil compositional effect as controls on subsurface oil biodegradation rates (Adams et al., 2006; Adams, 2008) is now widely utilized in industry. These models indicate that oil charge rates (the volumetric rate of oil charge to a filling reservoir), is a primary control on the quality of oil in a biodegrading oil reservoir. Larter et al. (2003), showed that in fact, the rates of charge and hydrocarbon destruction through biodegradation in heavy oil reservoirs, are commonly of a similar order of magnitude. Thus, small changes in charge rate can greatly affect the net levels of biodegradation seen in the oil field as fresh oil mixes with oil that is already biodegrading.

## Microbial processes and in-reservoir petroleum biodegradation

### Distinct patterns in microbial communities enriched in oil-degrading, methanogenic, and sulfate-reducing conditions

**Figure 10:**
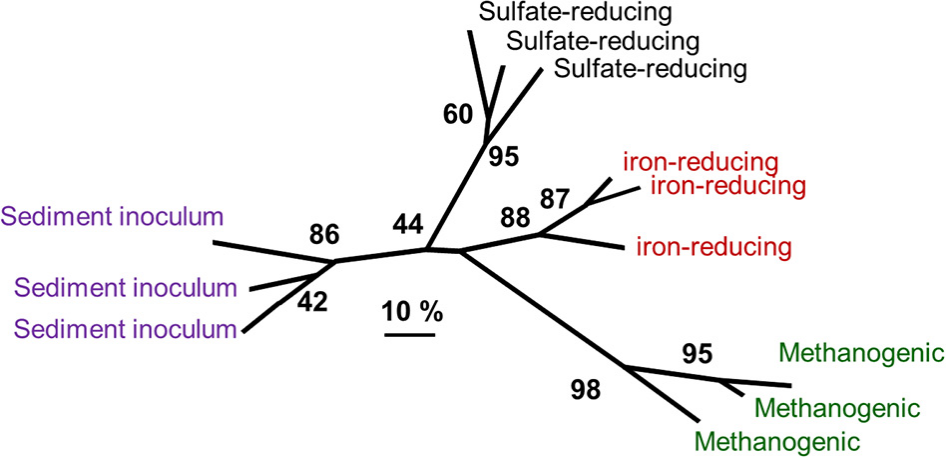
**Comparative analysis of bacterial community DGGE profiles from crude oil degrading microcosms incubated under different electron-accepting conditions and communities in the sediment used to inoculate the sediments**. Distinct communities were selected under different electron accepting conditions. The numbers at nodes represent the percentage of trees in which the group to the right of the node was recovered in datasets subject to bootstrap resampling (100 replicates) and gives an indication of the confidence that can be placed in the groups recovered. The scale represents 10% difference in the community composition based on Dice similarity of pair-wise comparisons of DGGE profiles.

Analysis of microbial communities from oil-degrading, methanogenic and sulfate-reducing microcosms inoculated with the same sediment and identical in every way except sulfate concentration has been reported recently by Gray et al. (2011) and Sherry et al. (2012). Comparison of denaturing gradient gel electrophoresis data from these oil-degrading systems and microcosms incubated under iron-reducing conditions shows that the bacterial communities enriched under different electron accepting conditions are distinct (Figure 10). *Deltaproteobacteria* from the *Syntrophaceae*, most closely related to bacteria from the genus *Smithella* were predominant in the methanogenic oil-degrading systems. This group is often detected at high frequency in methanogenic hydrocarbon-degrading systems (Dojka et al., 1998; Zengler et al., 1999; Bakermans and Madsen, 2002; Kasai et al., 2005; Allen et al., 2007; Shimizu et al., 2007; Ramos-Padrón et al., 2011; Siddique et al., 2011) and is implicated as the primary alkane utilizer in methanogenic crude oil alkane-degrading systems, at least in near surface oil contaminated sites (Gray et al., 2011) if not petroleum reservoirs (see Section Microbial Communities in Petroleum Reservoirs).

Contrary to expectation, known sulfate-reducing taxa were not enriched in oil degrading sulfate-reducing microcosms. During the period of most extensive sulfate-reduction coupled to crude oil alkane degradation, *Gammaproteobacteria* and *Firmicutes* were prevalent (Sherry et al., 2012) and represented by members of the genus *Marinobacterium* and members of the family *Peptostreptococcaceae* respectively. These organisms have been associated with hydrocarbon impacted environments in the past. Though considered aerobic, some *Marinobacterium* strains are known to be facultatively anaerobic (Kim et al., 2007). Moreover *Marinobacterium* has been identified in an oil-water separator tank from a Dutch oil field (van der Kraan et al., 2010) and a saline petroleum reservoir (Yuehui et al., 2008). *Firmicutes* have also been associated with anaerobic crude oil degrading systems (Gieg et al., 2008, 2010; Wang et al., 2011). Thiosulfate-reducing bacteria (TRB) from the families *Clostridiaceae* and *Peptostreptococcaceae* have been isolated from production waters in an onshore oil field in North-Eastern India, (Agrawal et al., 2010). It is therefore possible that the *Firmcutes* found at high frequency in the sulfate-reducing oil degrading microcosm are TRB rather than SRB. About 68–78% of the HS^−^ generated by sulfate reduction in sediments is re-oxidized to thiosulfate (Jorgensen, 1990) and endogenous generation of thiosulfate in the microcosms could potentially lead to the enrichment of thiosulfate-reducing hydrocarbon degraders. This is consistent with the observation that the sulfate- (and thiosulfate-) reducing archaeon *A. fulgidus* VC-16 degrades alkanes at 70°C with thiosulfate as an electron acceptor (Khelifi et al., 2014). In relation to the occurrence of thiosulfate reducers in petroleum reservoirs it is pertinent that many fermentative bacteria that have been identified in petroleum reservoirs are also capable of thiosulfate-reduction (Magot et al., 2000).

**Figure 11:**
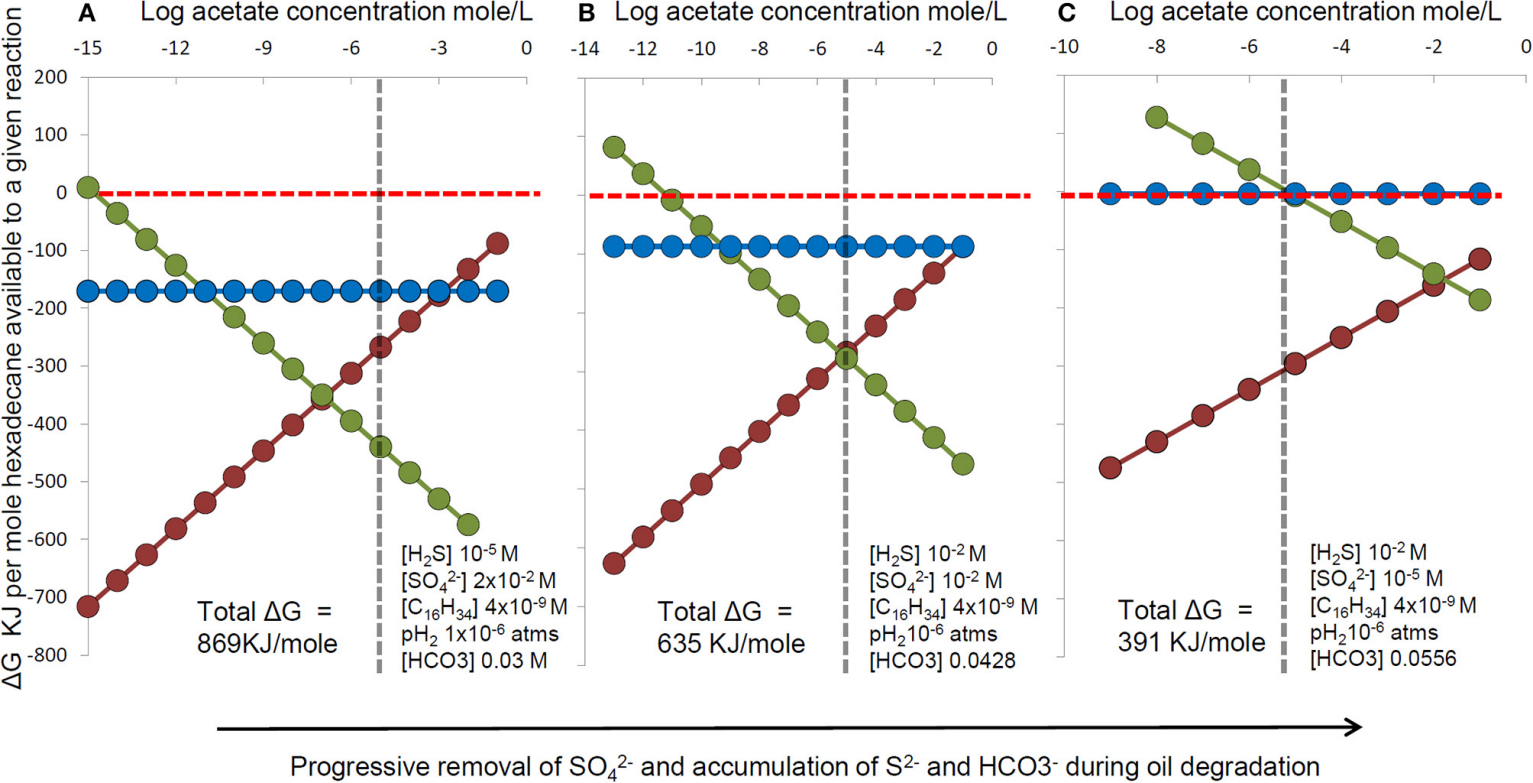
**The effect on thermodynamic yields of progressive changes in sulfate, sulfide and bicarbonate concentration during hexadecane degradation coupled to sulfate reduction (C_16_H_34_ + 12.25SO^2−^_4_ + 8.5H^+^ → 16HCO^−^_3_ + 12.25H_2_S + H_2_O)**. For each set of sulfate, sulfide and bicarbonate conditions **(A–C)** energy yields were calculated for a range of acetate concentrations and a fixed hydrogen partial pressure for a hypothetical microbial consortium comprising: (red circles and line) hexadecane fermentation to hydrogen and acetate (C_16_H_34_ + 16H_2_O → 8CH_3_COO^−^ + 8H^+^ + 17H_2_), (blue circles and line) hydrogen oxidation coupled to sulfate reduction (17H_2_ + 8.5H^+^ + 4.25SO^2−^_4_ > 4.25H_2_S + 17H_2_O), (green circles and line) Acetate oxidation coupled to sulfate reduction (8CH_3_COO^−^ + 8H^+^ + 8SO^2−^_4_ > 16HCO^−^_3_ + 8H_2_S). Calculations were performed using the concentrations indicated in each panel. All other conditions used in calculations were fixed e.g., 20°C, pH7 and fixed hexadecane (aqueous solubility) and hydrogen partial pressures pH_2_ (10^−6^ atms). The vertical dashed line represents a typical threshold level for acetate in sulfate reducing systems.

Nevertheless, re-oxidation of sulfide to thiosulfate still relies on sulfate reducers to produce sulfide in the first instance and their lack of strong enrichment may therefore result from oil being degraded syntrophically by consortia of hydrocarbon fermenting organisms with sulfate reducers simply consuming fermentation products. The partition of energy between the fermenters and sulfate-reducing syntrophs in such a system is dependent on *in situ* conditions such as sulfate concentration and accumulation of hydrogen and acetate for example as fermentation end products could result in preferential enrichment of the alkane fermenters relative to the sulfate-reducing terminal oxidizers in the system, leading to an apparent lack of enrichment of classical sulfate-reducers as has been observed (Sherry et al., 2012). We have conducted a thermodynamic analysis illustrating how energy partitioning between syntrophic hexadecane-degrading bacteria and sulfate-reducing terminal oxidizers changes with time as sulfate becomes depleted. As sulfate is depleted, sulfide and bicarbonate increase with hydrocarbon degradation. At acetate concentrations typical of sulfate-reducing sediments, the energy yield for alkane fermentation exceeds the energy available from hydrogen or acetate oxidation with sulfate (Figure 11) potentially leading to a situation that sulfate-reducing taxa are not predominant under conditions where extensive hydrocarbon degradation has occurred under sulfate-reducing conditions.

### Differences in hydrocarbons degraded, mechanisms and metabolites under methanogenic and sulfate-reducing conditions

**Figure 12:**
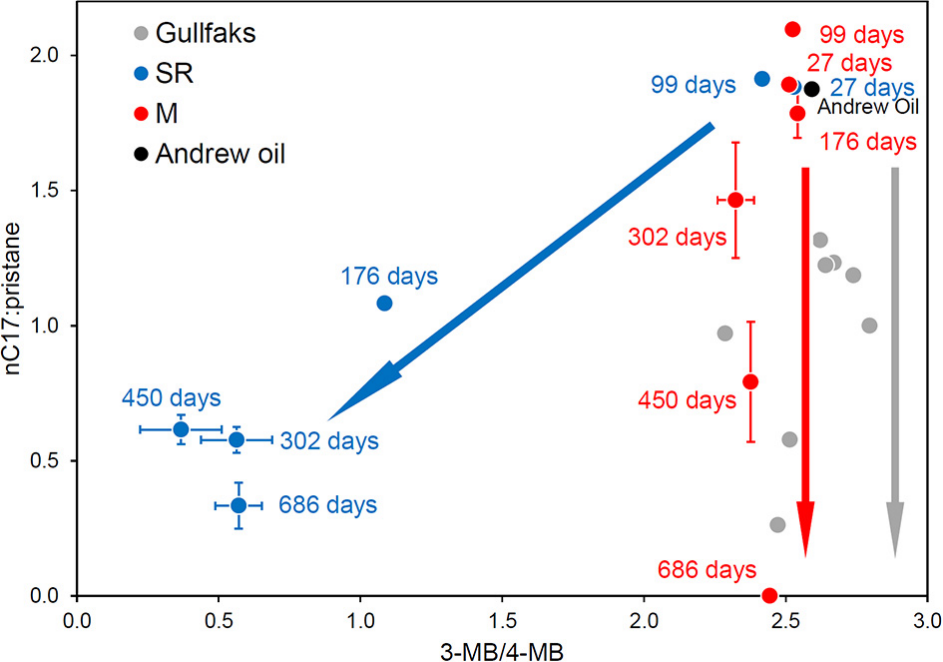
**Plot of the ratios of n-heptadecane to pristane against the 4-methylbiphenyl to 3-methylbiphenyl ratio from North Sea crude oil degraded in laboratory anaerobic microcosm experiments under sulfate-reducing (SR, blue) and methanogenic (M, red) conditions and field-degraded oils from the North Sea Gullfaks field (Gullfaks, gray)**. Error bars for the peak ratios are ±1 *SE* (*n* = 3). The laboratory methanogenic microcosm data and the field data plot along the same biodegradation trajectory with n-alkane degradation but no apparent aromatic hydrocarbon degradation, while the sulfate-reducing microcosm data show the concomitant degradation of n-alkanes and aromatic hydrocarbons under different electron accepting conditions.

**Figure 13:**
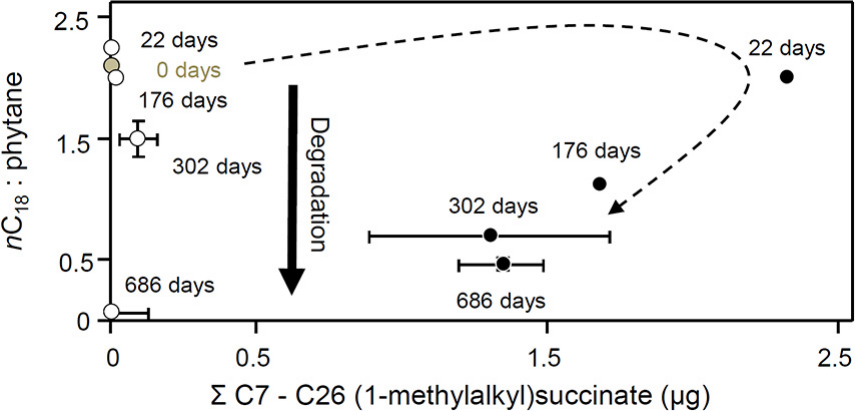
**Changes in nC18 to phytane ratio with total C7 to C26 (1-methylalkyl)succinates (μg) in methanogenic microcosms (open circles) and sulfate-reducing microcosms (filled circles) over 686 days of anaerobic hydrocarbon degradation**. Error bars, where shown, are ± one standard error of replicate microcosms where *n* = 3. The dotted arrow indicates the temporal changes in alkylsuccinate concentration under sulfate-reducing conditions.

Consistent with the prevalence of different bacterial populations in oil degrading systems under methanogenic and sulfate-reducing conditions, differences in the degradation of hydrocarbons e.g., biphenyl isomers (Figure 12) amongst other oil components, are seen under sulfate-reducing and methanogenic conditions (Townsend et al., 2003; Jones et al., 2008). This observation coupled with oil geochemistry from biodegraded reservoirs has provided one piece of evidence that in-reservoir oil degradation is driven by methanogenesis (Jones et al., 2008; Figure 12). Interestingly, in oil-degrading, sulfate-reducing laboratory incubations, considerable accumulation of alkylsuccinates, key products of fumarate addition reactions, was observed. By contrast during methanogenic oil degradation these metabolites were present only at levels similar to control incubations without oil, and levels detected in the sediment used to inoculate the microcosms (Figure 13; Aitken et al., 2013). Similar observations have been reported for other methanogenic oil degrading systems (Gieg et al., 2010; Zhou et al., 2012) suggesting that under methanogenic conditions crude oil alkanes may be activated by a different mechanism. Alternatively the kinetics of alkylsuccinate consumption in the methanogenic alkane-degrading systems may be more rapid leading to lower concentrations of these key intermediates. Interestingly, it has been shown that methanogenic toluene degradation is most likely initiated by fumarate addition, though in this case the corresponding benzylsuccinates accumulated transiently (Beller and Edwards, 2000; Fowler et al., 2012). Quantification of alkylsuccinate synthase genes (*assA*) also demonstrated that these only increased in abundance during sulfate-driven crude oil alkane degradation and not in methanogenic oil degrading systems. This suggested that organisms harboring *assA* genes were not enriched under methanogenic conditions. An alternative mechanism for alkane activation under methanogenic conditions was supported by recent metagenomic analysis of a methanogenic hexadecane-degrading enrichment culture (Embree et al., 2013). Embree et al. (2013) did not identify *assA* genes in the metagenome data, however reanalysis of the data demonstrated that *assA* genes were present, but they had been annotated as pyruvate-formate lyase genes by the automated annotation pipeline used in the initial analysis of the metagenome (Tan et al., 2014). Not only this, but reanalysis of the metatranscriptome data from the study of Embree et al. (2013) showed that the gene was up-regulated during alkane degradation (Tan et al., 2014). Moreover, a defined mixed culture of *Desulfatibacillum alkenevorans* AK-01, which activates alkanes via fumarate addition, and *Methanospirillum hungatei* JF-1 converts alkanes to methane (Callaghan et al., 2012). It is thus clear that the landscape of anaerobic alkane activation is not straightforward and it may be difficult to associate alkane degradation under particular electron-accepting conditions, with a specific alkane activation mechanism.

Hydrocarbon addition to fumarate which involves formation of substituted succinates as catabolic intermediates is the best characterized mechanism for activation of hydrocarbons in the absence of oxygen. This mechanism has been observed under a wide range of electron accepting conditions and represents a central paradigm in anaerobic hydrocarbon degradation. However, alternative pathways have been proposed. For instance sub-terminal carboxylation of alkanes at C-3, with the formation of 2-ethyl fatty acids with subsequent removal of the two terminal carbons has been suggested (Aeckersberg et al., 1998; So et al., 2003; Boll and Heider, 2010). This results in formation of C-odd fatty acids from C-even alkanes (and vice versa) by the sulfate-reducing strain *Desulfococcus oleovorans* Hxd3. Interestingly So et al. (2003) were unable to detect 2-ethyl fatty acid intermediates and likewise they were not detected in the acid fraction from the crude-oil degrading methanogenic microcosms investigated by Aitken et al. (2013). In addition, there is a yet undetermined mechanism for the formation of C-even cellular fatty acids from C-even alkanes by a denitrifying bacterium *Pseudomonas balearica*. This organism utilizes C15 -C18 alkanes as substrate but does not appear to use fumarate addition for initial alkane activation (Grossi et al., 2008).

Anaerobic hydroxylation of an alkyl carbon in ethylbenzene by a molybdenum-containing enzyme has also been observed (Johnson et al., 2001). Hydroxylation of alkanes is considered less likely due to the higher C-H bond dissociation energy of alkanes (400 kJ/mol) compared to the C-H bond dissociation energy of C-2 of the alkyl side-chain of ethylbenzene (355 kJ/mol) (Boll and Heider, 2010). Alkane C-H bond dissociation energy reduces slightly with alkane chain length and is slightly lower for C-2 of n-alkanes and secondary or tertiary carbon atoms, but such reactions have only been considered feasible when a high potential electron acceptor such as nitrate or FeIII is used. This is consistent with the observation that nitrate- and iron-reducers are capable of hydroxylation of the methyl group of cresol, but typically sulfate-reducers degrade cresol by fumarate addition (Boll and Heider, 2010). Nevertheless, there is no *a priori* reason that hydroxylation of an aliphatic carbon with a C-H bond dissociation energy greater than 355 kJ/mol is not possible and it now seems that alkane activation by *Desulfococcus oleovorans* may not proceed by initial carboxylation at C-3 of the alkane but instead by hydroxylation at C-2 followed by oxidation of the hydroxyl group and carboxylation at C-3 of the methylalkyl ketone (Callaghan, 2013).

## Microbial communities in petroleum reservoirs

Most data on microbial communities in petroleum reservoirs come from produced waters and there are almost no data on sediments recovered from petroleum reservoirs by coring (Magot, 2005; Head et al., 2010). The nature of produced waters, and issues as to whether or not water injection for secondary recovery has been practiced leads to difficulties in assigning the provenance of organisms cultured from petroleum reservoirs (Stetter et al., 1993; Magot, 2005). A substantial proportion of the organisms identified in culture independent analyses of produced waters from petroleum reservoirs are therefore considered to be non-indigenous organisms that have grown in the production infrastructure (Orphan et al., 2000; Grabowski et al., 2005). To date there have been few studies which have focused specifically on the microbial communities present in biodegraded petroleum reservoirs (Grabowski et al., 2005; Sette et al., 2007; Dahle et al., 2008; de Oliveira et al., 2008; Hallmann et al., 2008; Hubert et al., 2012; Kobayashi et al., 2012). In some of these there were apparently no differences in the communities present in degraded and non-degraded reservoirs (Sette et al., 2007; de Oliveira et al., 2008) though the relative abundance of taxa detected in 16S rRNA gene clone libraries did differ in one study (Sette et al., 2007).

**Figure 14:**
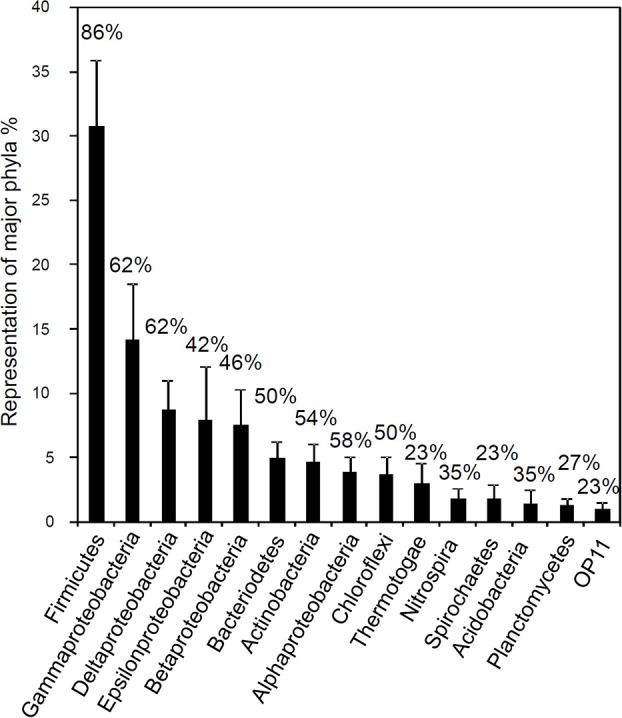
**Frequency distribution of 16S rRNA sequences (classified into major phylogenetic groups) recovered in clone libraries from hydrocarbon impacted environments**. Bars correspond to average percent representation of major phyla (1× *SE*) based on a survey of 26 bacterial clone libraries. Values shown above the columns indicate the percentage of studies in which the phylum was identified. Modified from Gray et al. (2010).

A recent broad survey of microbial community data from a range of oil and hydrocarbon-impacted anoxic environments has demonstrated that the group of organisms found most frequently, and at the highest relative abundance is the *Firmicutes* followed by the *Gamma*-, *Delta*- and *Epsilonproteobacteria* (Gray et al., 2010; Figure 14). This survey included data from systems that contained both biodegraded and non-biodegraded oil and also some examples of enrichment cultures and microcosm studies where active hydrocarbon degradation was occurring. The analysis indicated that *Smithella* spp. may play a central role in methanogenic crude oil alkane degradation in anoxic environments lacking any alternative electron acceptors (Head et al., 2010; Gray et al., 2011; Cheng et al., 2013a). However, although a number of surveys report the detection of *Syntrophaceae* related to *Smithella* and *Syntrophus* in petroleum reservoir systems (Gray et al., 2011 and reference therein) the majority of studies relevant to biodegraded oil fields indicate that they are detected at low frequency (Gieg et al., 2008; Wang et al., 2011; Kryachko et al., 2012; Mbadinga et al., 2012). Crude oil and hydrocarbon-degrading enrichments inoculated with oil field waters reveal that other organisms may provide the key function of alkane fermentation, especially in high temperature systems (Gieg et al., 2010; da Cruz et al., 2011; Wang et al., 2011; Mbadinga et al., 2012). In the majority of these cases *Firmicutes*, predominantly *Clostridia* were detected at highest frequency (19–71% of 16S rRNA gene clones; (Gieg et al., 2010; Wang et al., 2011; Mbadinga et al., 2012). In one case *Bacillus* and *Acinetobacter* were predominant (da Cruz et al., 2011) although the medium used in this study contained 8 mg of yeast extract in each 40 ml microcosm as well as 30 mg of oil, of which only a small fraction would have been biodegraded over the time course of the experiments (da Cruz et al., 2011). Control incubations with no oil were also run in this study, however the composition of the microbial communities in the control incubations was not reported (da Cruz et al., 2011).

The significance of *Firmicutes*, especially those related to *Clostridia*, in anaerobic hydrocarbon degradation associated with petroleum reservoirs seems increasingly likely. *Firmicutes* identified in methanogenic oil degrading systems include organisms related to known syntrophic acetate oxidizing bacteria such as *Thermacetogenium* and *Moorella* (Gieg et al., 2010; Mbadinga et al., 2012). The presence of syntrophic acetate oxidation to H_2_ and CO_2_ was linked to a shift in methanogenic communities from acetoclastic to hydrogenotrophic methanogens with an increasing number of transfers of a methanogenic hexadecane-degrading enrichment, inoculated with water from the Shengli oilfield, China (Cheng et al., 2013c). This is particularly interesting given the prevalence of hydrogenotrophic methanogenesis by CO_2_ reduction in petroleum reservoirs (Head et al., 2010) and the fact that syntrophic acetate oxidation has been implicated as a central process in methanogenic crude oil biodegradation in some systems (Jones et al., 2008; Gray et al., 2011). The significance of *Firmicutes* is also suggested by the increase in concentration of anteiso branched phospholipid fatty acids detected in biodegraded oils (Hallmann et al., 2008). In higher temperature enrichments (55 to 74°C; Gieg et al., 2010; Mbadinga et al., 2012) thermophilic taxa (e.g., *Thermodesulfobiaceae* within the *Clostridia*, and *Thermotogae*) were also enriched opening up the possibility that a relatively broad range of organisms may have the capacity for anaerobic degradation of different crude oil components. Interestingly, the organisms selected in 55°C enrichment from an Alaskan oil reservoir were also detected in produced waters from the same reservoir (Duncan et al., 2009; Gieg et al., 2010). The microbial communities enriched in high temperature oil degrading systems appear to be quite different from those observed under mesophilic conditions (Gray et al., 2010). Gieg et al. (2010), for example identified organisms from the *Thermotogales* (closest relative *Thermotoga elfii*), *Deferribacterales* (related to *Flexistipes* spp.), *Synergistales* (related to *Anaerobaculum hydrogeniformans* OS1) and *Thermoanaerobacterales* (related to *Thermacetogenium phaeum*, a syntrophic acetate oxidizer from the *Firmicutes*). A high temperature (55°C) methanogenic enrichment culture which degraded hexadecane has also been found to be dominated by a novel deep branching bacterial group related to *Thermotoga* spp. and has been designated the Shengli Cluster (Cheng et al., 2013b). It is interesting to speculate that these organisms might represent an entirely new group of thermophilic hydrocarbon-degrading syntrophs.

### Microbial communities in heavily biodegraded reservoirs

When one examines the composition of microbial communities from analysis of produced waters from the most heavily biodegraded oil reservoirs, the picture that emerges is somewhat different. Microbial communities associated with waters from heavy oil fields in the Western Canadian Sedimentary Basin and Oil Sands reservoirs appear to harbor communities that are dominated by *Epsilonproteobacteria* (Grabowski et al., 2005; Hubert et al., 2012). It has been suggested that this dominance is artifactual caused by selectivity of the primers used for 16S rRNA gene analysis (Grabowski et al., 2005), however, more recent work points to the genuine dominance of *Epsilonproteobacteria* in these systems (Hubert et al., 2012). *Epsilonproteobacteria* were also detected as abundant members of coal bed microbial communities (US Patent 2010/0047793 A1, 2010). The fact that the petroleum in heavy oil reservoirs has low levels of saturated hydrocarbons like *n*-alkanes and contain higher levels of aromatic hydrocarbons and more polar aromatic components such as benzothiophenes, might explain why the microbial communities in biodegraded heavy oil reservoirs appear to be distinct. At present the specific role of the resident microbiota in heavy oil biodegradation is unknown, For instance, many *Epsilonproteobacteria* are putative chemoautotrophic, nitrate-reducing, sulfide oxidizers and some may be responsible for sulfide oxidation in sour petroleum reservoirs (Gevertz et al., 2000; Hubert, 2010). However, they may be autotrophic sulfide oxidizing autotrophs and heterotrophs utilizing a range of electron acceptors (Campbell et al., 2006). *Sulfuricurvum kujiense* is regarded as a sulfide oxidizing chemoautotroph but the type strain YK-1 can obtain energy for growth by oxidizing reduced, probably organic, sulfur in crude oil (Kodama and Watanabe, 2003, 2004). This could explain the abundance of *Sulfuricurvum* in highly biodegraded oil reservoirs such as the Canadian oil sand reservoirs, where the oil has a relatively high sulfur content (Strausz and Lown, 2003). Moreover the oil-water transition zone between oil sands reservoirs and underlying aquifers can exhibit gradients of a range of aromatic hydrocarbons and sulfur heterocycles such as dibenzothiophenes and such compositional gradients are indicative of active biodegradation in transition zones in oil sands (Head et al., 2003; Hubert et al., 2012).

Oxidation of reduced sulfur compounds *in situ* may be driven by nitrate reduction (Hubert, 2010) or reduction of metal species (Aller and Rude, 1988). Indeed some *Arcobacter* and *Sulfurospirillum* spp. are capable of iron or manganese reduction (Thamdrup et al., 2000). *S. kujiense* strain YK-1, like some other *Epsilonproteobacteria*, can grow under microaerophilic conditions (Kodama and Watanabe, 2003, 2004) and use oxygen as an electron acceptor (Campbell et al., 2006). It is therefore also possible that small amounts of oxygen introduced from meteoric water into aquifers associated with shallow heavily degraded oil sands reservoirs, could provide an electron acceptor for sulfide or organic sulfur oxidation.

Some studies suggest *Epsilonproteobacteria* might play a role in syntrophic anaerobic hydrocarbon-degrading communities. Assimilation of ^13^C into nucleic acids from an epsilonproteobacterium during syntrophic degradation of ^13^C-labeled benzene in sulfate-reducing enrichment cultures has been observed, and in fact the highest degree of ^13^C enrichment was observed for the *Epsilonproteobacteria* (Herrmann et al., 2010). It is possible, therefore, that this organism may contribute to benzene fermentation to acetate, H_2_ and CO_2_ that are subsequently used by the sulfate-reducers in the system. *Sulfurospirillum* spp. are metabolically versatile and can ferment a wide variety of organic compounds (Luijten et al., 2003) and were among the fermentative heterotrophs isolated from a subsurface coal deposit (Fry et al., 2009). *Sulfurospirillum* spp. were also isolated from the Pelican Lake oil reservoir in northern Alberta (Grabowski et al., 2005). *Arcobacter* spp. utilize acetate under anoxic conditions (Thamdrup et al., 2000; Fedorovich et al., 2009; Webster et al., 2010) and several *Epsilonproteobacteria* can use H_2_ as an electron donor (e.g., Gevertz et al., 2000; Kodama et al., 2007), including an acetogenic *Arcobacter* abundant in the Pelican Lake reservoir bacterial community (Grabowski et al., 2005).

### Putatively aerobic organisms and functional genes in petroleum reservoirs

Increasingly in the literature on hydrocarbon-containing subsurface reservoirs there are reports of organisms conventionally considered to be aerobic (or in some cases facultative) heterotrophs e.g., *Bacillus* spp., *Acinetobacter* spp. and *Pseudomonas* spp., particularly in heavy, biodegraded oil reservoirs such as the Alberta oil sands (Orphan et al., 2000; da Cruz et al., 2011; Li et al., 2012; Zhang et al., 2012; An et al., 2013; Meslé et al., 2013). This raises questions about the anaerobic/methanogenic origin of heavy oil that has prevailed for a number of years (Head et al., 2003). What then, does the occurrence of these apparently aerobic organisms indicate about the biodegraded petroleum systems where they occur?

There are a number of possible explanations for these observations.

- Samples were exposed to oxygen during sampling, transport and storage allowing growth of aerobic organisms initially present at low abundance.
- Oxygen was supplied externally to the reservoir by meteoric water.
- A “cryptic” aerobic community that uses *in situ* generated oxygen may be present in some petroleum systems.
- The aerobes detected are in fact capable of anaerobic metabolism.

The first of these, oxygen exposure and outgrowth of populations of aerobes initially present at low abundance, is certainly a possibility given the technical difficulties in obtaining high quality samples from operational petroleum systems. However, one might expect, for example, that a suite of samples from the same core would be equally likely to be exposed to oxygen and therefore all samples should contain this aerobic microbial community signal. This does not seem to be the case and communities exhibiting primarily aerobic signatures have been found adjacent to samples harboring communities with a predominantly anaerobic character (An et al., 2013).

If aerobic organisms are indeed residents of petroleum reservoirs, where might oxygen come from in deep subsurface sediments? Oxygen could be delivered to oil sands reservoirs in meteoric water, as envisaged in the classical model of in-reservoir petroleum biodegradation (Palmer, 1993). This ingress might be facilitated by the relatively shallow depth of reservoirs e.g., the Western Canada Sedimentary Basin, or by faults and fractures providing preferential migration paths for water, delivering oxygen without consumption by microorganisms or reaction with minerals. Repeated glaciation-deglaciation cycles in the oil sands area, over the last 20,000 years, could also have potentially delivered cold-oxygen containing waters to shallower reservoirs and this would be somewhat facilitated by isostatic rebound, pulling water into the sediment as ice sheet loading decreased, in effect, transporting water by “isostatic pumping.”

A second possible reason for aerobes in deep subsurface sediments is the occurrence of a “cryptic” aerobic community using oxygen generated *in situ*. Mechanisms are known, or proposed, whereby oxygen may be generated microbially by dismutation of chlorate, nitrate or nitrogen oxides (Achenbach et al., 2006; Ettwig et al., 2012) however these species are not typically found naturally in petroleum reservoirs, though they may be present in reservoirs subject to e.g., nitrate injection for souring control (Hubert, 2010). There is a considerable literature on radiolysis of water generating hydrogen to fuel the deep biosphere (Lin et al., 2005). However, much less consideration has been given to the oxygen species that are also generated by radiolytic splitting of water (Bjergbakke et al., 1989; Draganic, 1991). Interestingly, the obligate aerobe *Deinoccocus radiodurans* only grows anaerobically if the culture is exposed to ionizing radiation presumably making use of oxygen generated from radiolysis of water. Although oxygen generation *in situ* has the capacity to support an aerobic microbial community, the amount generated may not be sufficient to support the degree of oil degradation required to produce heavy oil such as found in the Western Canada sedimentary basis.

If one assumes a rate of water radiolysis of 2 × 10^−8^ nanomoles/dm^3^/s (based on a range of 1.5 to 4.5 × 10^−8^ nanomoles/dm^3^/s from data in Lin et al., 2005) then this could generate H_2_ at rate of 2 × 10^−8^ nanomoles/dm^3^/s and O_2_ at a rate of a rate of 1 × 10^−8^ nanomoles/dm^3^/s. Thus, for 1 m^3^ of sediment, oxygen could be generated at 10^−5^ nanomoles/s or 3 × 10^−7^ moles/y.

Based on oil compositional gradients, areal oil degradation fluxes at the OWTZ of reservoirs containing heavy oil have been estimated at around 10^−5^ to 10^−6^ kg/m^2^/y (Larter et al., 2003). Given that oil is about 85% carbon this results in a carbon flux of 8.50 × 10^−3^ to 8.50 × 10^−4^ g C/m^2^/y or 7.1 × 10^−4^ moles C/m^2^/y. On this basis we can estimate a fractional rate for aerobic oil degradation (*F*_aerobic_ = aerobic degradation/total degradation). To convert volumetric rates to fluxes we can assume that a given depth of water aquifer, underlying the OWTZ, contributes to the oxygen fed to it. Thus, for 1 m of water leg contributing oxygen, *F*_aerobic_ = 3 × 10^−7^/7.1 × 10^−4^ = 3 × 10^−4^ or 0.042%. For 100 m of water leg *F*_aerobic_ = 3 × 10^−5^/7.1 × 10^−4^ = 3 × 10^−2^ or 4.2% of the total degradation flux. These are very crude calculations, but the values obtained are likely to be overestimates and suggest complete aerobic oxidation of hydrocarbons by this mechanism is not significant. Radioloysis of water generates a number of different oxygen species not just O_2_ (Bjergbakke et al., 1989; Draganic, 1991) and these are more reactive than oxygen and probably not available to microbial communities. It is also unlikely that all of the oxygen generated from the aquifer underlying a reservoir would reach the OWTZ reinforcing the conclusion that radiolysis of water could only make a small contribution to aerobic oil degradation in the subsurface. It is interesting to speculate, however, that trace levels of oxygen are not used to completely mineralize organic compounds, but instead for an initial activation of the hydrocarbon molecules, with subsequent anaerobic metabolism. Such dysaerobic activation and anaerobic metabolism (DAAM), could then be much more significant, but still a minor contributor to net biodegradation rates. Moreover, during biodegradation of light oil, high levels of labile substrates are present in the oil and anaerobes might effectively compete with aerobes which are limited by very low oxygen levels. However, in heavy oil, electron donors and carbon sources may be more limiting and aerobes will be able to compete more effectively even if oxygen levels are very low which may lead to a situation where aerobes might dominate the habitat due to the higher growth yields that can be achieved with oxygen as an electron acceptor. It should however also be considered that maintenance energy requirements for aerobes are also typically higher than for anaerobes and thus at very low oxygen concentrations they may not achieve high growth yields.

The bulk, molecular and gas geochemistry of heavy oilfields is also a major bounding constraint, with the gases associated with heavy oilfields showing very dominant methane and minor CO_2_ content and carbon isotopic signals inconsistent with large-scale aerobic processes. Thus, in the Albertan heavy oil fields (Adams, 2008; Adams et al., 2013a), the carbon isotopic signatures of associated carbon dioxide in equivalent reservoirs are much heavier (δ ^13^C CO_2_ of −10 to +20 per mil typically), than that expected by direct aerobic oxidation of source crude oils. Oils sourced from the Exshaw and Gordondale Fm. in this area, have whole oil δ^13^C signatures of around −30 to −31 per mil (Adams et al., 2013a), and they would be expected to produce CO_2_ as the dominant gaseous end product, and this would have a similar carbon isotopic signature to the source oils, if formation of heavy oil in the oil sands was predominantly a product of aerobic biodegradation. Thus, the gas geochemistry of the heavy oils and oil sands is not consistent with aerobic biodegradation being a very significant formative process. The dominant methane, subordinate CO_2_ and often very heavy carbon isotopic signatures of the CO_2_ observed in heavy oils and oil sands reservoirs, is consistent with anaerobic and specifically methanogenic processes (Jones et al., 2008).

The final possibility is that putative aerobes observed in petroleum reservoirs and other deep subsurface sediment environments, are in fact facultative organisms capable of anaerobic metabolism. *Pseudomonas* spp. for example, classically thought of as catabolically versatile aerobes or facultative aerobes that utilize nitrate and other oxidized nitrogen species as alternative electron acceptors to oxygen, are probably more cosmopolitan in their use of electron acceptors than commonly considered. At least one *Pseudomonas* sp. (*Pseudomonas aeruginosa* strain KRP1) is known to use solid phase anodes in a microbial fuel cell (MFC) as an electron acceptor and it achieves this through the mediation of phenazine electron shuttles that deliver electrons from the cell to the anode of the MFC (Rabaey et al., 2005).

Potentially putative aerobes may even be able to grow as syntrophs in partnership with methanogens. This tantalizing possibility has recently been raised by a study of a methanogenic, crude oil-degrading enrichment culture inoculated with oil reservoir production water (Berdugo-Clavijo and Gieg, 2014). In this study, a methanogenic oil degrading consortium, dominated by *Smithella* sp., a putative alkane fermenting organism, and a range of acetoclastic and CO_2_-reducing methanogens, was inoculated into anoxic sand columns containing residual oil, and incubated in an anoxic chamber for over 300 days. The sand columns actively degraded crude oil hydrocarbons and generated methane. When the microbial communities in the sand columns were analyzed, the composition of the microbial communities had changed considerably with CO_2_-reducing methanogens predominating in the community. However, the most intriguing observation was that the most highly represented bacterial taxon was a *Pseudomonas* sp. (Berdugo-Clavijo and Gieg, 2014). While the authors were measured in their interpretation that this *Pseudomonas* sp. might represent the syntrophic hydrocarbon-degrading partner of the methanogens in the culture, the results do raise the intriguing possibility that some *Pseudomonas* spp. have evolved to occupy a niche whereby they are active under highly reducing conditions. Their detection in an increasing range of anoxic environments may relate to their capacity to thrive in these environments rather than representing mere aerobic contaminants. This view is strengthened by anaerobic isolation of *Pseudomonas* sp. GZ1 (Guo et al., 2008). GZ1 was isolated using the Hungate method and shown to ferment organic compounds to hydrogen and may therefore have the capacity to grow in a syntrophic partnership with methanogens for example. Thus, we speculate, that at least some of these putative aerobes are likely, putative no more, and are in fact most likely anaerobic partners in the biodegradation process itself. It would appear that the study of anoxic environments exhibiting petroleum biodegradation continues to overturn conventional learnings of classical microbiology.

## Geological learnings and practical applications

While much of what is presented above is of importance in understanding the fundamental biogeochemistry of heavy oil reservoirs, it is not without practical significance. It is well know that one of the major impacts of biodegradation on petroleum is to increase oil viscosity primarily by destruction of low molecular weight compounds (Adams et al., 2008; Larter et al., 2008). Biodegradation of crude oil driven from a basal biodegradation zone produces vertical viscosity gradients. These vertical and complementary lateral oil viscosity gradients have major impact on oil production strategies and production rates (Larter et al., 2008). In many reservoirs steps or breaks in the compositional and oil viscosity profiles are common, reflecting reservoir compartmentalization through baffles, barriers or even faults (Fustic et al., 2011). Characterization of vertical and lateral viscosity gradients and intra-reservoir baffles and barriers using geochemistry is now a major commercial application in the heavy oil industry as it allows better producing, less viscous zones in the reservoir to be targeted for production (Sereda and James, 2014). Modeling approaches have also been developed to facilitate the identification of production sweet spots (Larter et al., 2003, 2008). Similar effects are seen in more conventional recovery processes, and it can probably be stated that biodegradation ubiquitously produces fluid property gradients in heavy oil reservoirs and these have a major impact on production strategies and efficiencies.

In-reservoir, crude-oil biodegradation is not therefore merely a phenomenon of academic significance, it impacts the occurrence, distribution and properties of the most abundant form of liquid petroleum on planet Earth, as well as being responsible for generation of a significant fraction of the natural gas that we produce today (Milkov, 2011).

## Impact on energy recovery and carbon management

The discovery of MADCOR raises many possibilities for reduced emission processes for energy recovery from heavy oil. This is because methane is quantitatively produced from biodegradation of hydrocarbons, utilizing water as a co-reactant, with molecular hydrogen gas being the principal intermediate (Jones et al., 2008). If the process could be dramatically accelerated by engineering, hydrogen generation and recovery *in situ*, could provide the basis of a truly green energy recovery process (Larter et al., 2012). In oil reservoirs, acetoclastic methanogenesis seems subordinate and most methanogenesis (>80%), results from reduction of CO_2_ with hydrogen. Dolfing et al. (2008), demonstrated that, for the crucial steps, a low hydrogen partial pressure (<4 Pa/ 4 × 10^−5^ atm) is required for thermodynamic feasibility and this is achieved naturally in oilfields by methanogens using hydrogen for CO_2_ reduction. So, while it is theoretically feasible to recover hydrogen from biodegrading oilfields, the low hydrogen concentrations present when the process is taking place actively, would render this difficult to engineer as a large scale process. To achieve this would require the development of dynamically managed recovery systems. These have been proposed, but would be a considerable technical challenge (Larter et al., 2012). The prospect that CO_2_ produced when alkanes are degraded, with the formation of hydrogen, could be selectively sequestered in-reservoir by manipulating formation water properties is also an attractive element of such a process, but this too, on a reservoir scale, would be a significant technical achievement.

**Figure 15:**
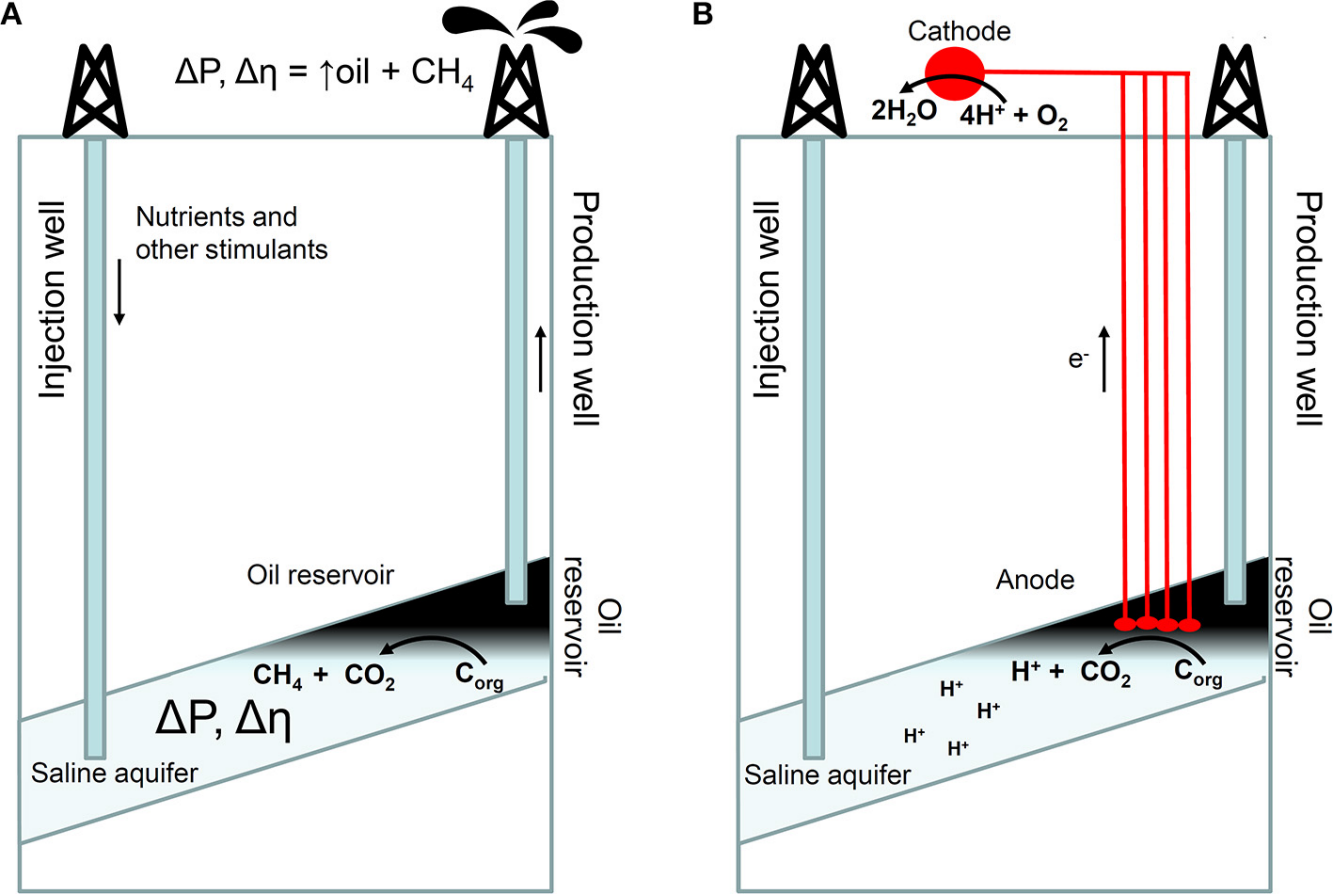
**Proposed systems for energy recovery from stranded, residual oil in petroleum reservoirs. (A)** Enhanced oil and gas recovery by stimulation of methanogenic oil biodegradation and **(B)** a bioelectrochemical system for the direct recovery of energy, as electricity from residual and heavy oil. Organic carbon (C_org_), Increase in pressure from gas generation (ΔP), decrease in oil viscosity due to gas dissolving in oil (Δη).

Accelerated methane production through nutrient addition to heavy oilfields is feasible today, but with low natural gas prices in North America resulting from recent increases in shale gas recovery, there is currently little business interest in such schemes. However, increased oil production together with gas is being considered as a biologically assisted engineering recovery process following on from conventional oil recovery (Figure 15A). An alternative to terminal methane production could be use of methanotrophs (methane consuming organisms) to convert produced methane to higher molecular weight hydrocarbons, other chemical species or methanol, directly or indirectly. Biological approaches that use methanotrophs to produce long-chain hydrocarbons have been identified as a priority by the United States government (Chu and Marjumbdar, 2012). Thus, a biological: heavy oil to methane to heavier hydrocarbons or other fuels using methanogenic and methanotrophic routes is possible but seems a stretch at the present.

Are alternate futures possible therefore? The heavy oil and bitumen resource worldwide, represents a vast chemical resource that could drive various chemical or electrochemical processes *in situ* or *ex situ* that might be used for low emission energy recovery including being a source of hydrogen or electrons via a large scale biolelectrochemical system (Figure 15B). Such a biotechnological application is feasible in principle but implementation on a reservoir or even basin scale is not trivial. A “pure” bioelectrochemical system will also need to contend with issues such as low power output and high internal electrical resistance in a large scale system. However, using the reducing power linked to an accessory power source to synthesize intermediate energy vectors such as in a microbial electrolysis cell (Cheng and Logan, 2007) or to charge batteries (Xie et al., 2013) could be considered as hybrid technologies to overcome some of these fundamental hurdles.

**Figure 16:**
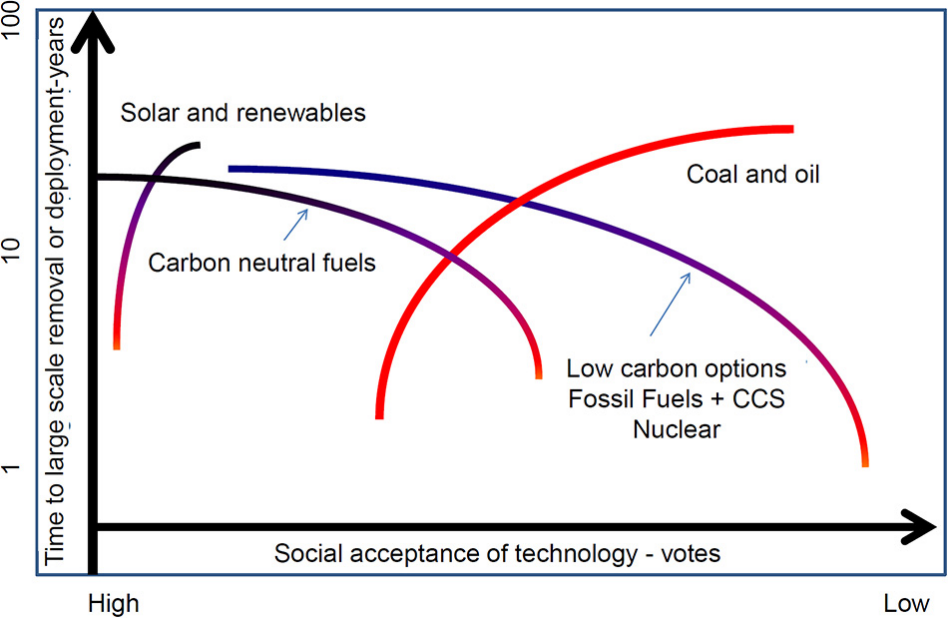
**The carbon management and energy universe; Our best estimate of the likely development routes for different energy resources over the next few decades**. The y-axis indicates the timescale to large-scale removal (reduction to 20% of market share, or deployment of a technology type (expansion beyond 20% of market share), and on the x-axis social acceptance and thus deployability of the technology type.

History tells us it is impossible to predict the future over timescales of more than a few months or years. Our best and simplistic estimate of development routes for energy resources over the next few decades is based on both technological readiness and social and political acceptance (Figure 16). Coal and oil use will continue for several decades, but their usage will need to decline substantially before 2050, when it is estimated that industrialized country emissions need to fall 10% below current levels. Today, coal and oil use is socially acceptable as sensible energy pricing and charges for emitted carbon dioxide are politically difficult to implement, but without emissions reduction technology in place, they will likely become socially unacceptable and decline in use. Transition away from traditional fossil fuels will initially involve lower carbon fuel options including greater use of natural gas and increased coupling of carbon capture and storage technologies to the traditional fossil mix, plus inevitable growth in next generation nuclear electricity generation. Carbon capture and storage, and nuclear are currently viewed as only marginally desirable by society, but they will have to grow in the next decade if 2050 emissions targets and a more sustainable energy supply is to be achieved. Solar and renewable energy will also continue to grow in significance, but is unlikely to represent more than 20–30% of the energy base by the same date. However, economically profitable incumbent industries resist change. Carbon dioxide capture and storage probably then provides the first step on the ladder of transition, but carbon neutral fuels (Zeman and Keith, 2008; Graves et al., 2011; Lackner et al., 2012), would also provide an effective strategy whereby carbon dioxide captured from the air is reduced by hydrogen (from low carbon energy sources such as hydroelectric or nuclear electricity), producing the range of transportation fuels that we have today. This techno-economic-finesse would potentially allow the petroleum industry further space for rapid transition, maintaining a hydrocarbon economy while ceasing to produce additional fossil carbon. While much development work still needs to be done, and there is no general agreement on a feasible price for the cost of carbon dioxide capture from the air, it has been suggested that an air capture-based, carbon dioxide to fuel cycle might be a suitable topic for a large strategic focused research program carried out along the lines of the Manhattan or Apollo projects (Broecker, 2013). Where might biotechnology play a role here (beyond plausible Nth generation biofuels, of course)? While current air capture of carbon dioxide schemes involve liquid, solid carbon dioxide sorbent phases (e.g., Stolaroff et al., 2008), biological capture schemes using novel high pH water chemistries and organisms together with novel bioreactors and fermentative recycling of the generated biomass to methane have been proposed recently (Strous, 2014). This is an area that that has such great potential, albeit with substantial engineering and economic obstacles to overcome, that large growth in this area would be expected. However, the social acceptance of air captured CO_2_ and carbon neutral fuels remains low (Figure 16), largely because the public is mostly unaware of such possibilities. We suggest that this will be an increasingly important route for investigation as a viable solution to our carbon predicament. In the century of biology, it seems inevitable that biotechnological options for energy production linked to reduction in atmospheric CO_2_ concentrations will be a major research effort.

## Author contributions

Ian M. Head and Stephen R. Larter conceived and wrote the manuscript, Neil D. Gray edited and revised the paper, provided unpublished data and provided intellectual input to many of the ideas developed in the manuscript.

### Conflict of interest statement

The Review Editor Marc Strous declares that, despite being affiliated to the same institution as the author Stephen R. Larter, the review process was handled objectively and no conflict of interest exists. The authors declare that the research was conducted in the absence of any commercial or financial relationships that could be construed as a potential conflict of interest.
